# A computational analysis of the role of integrins and Rho-GTPases in the emergence and disruption of apical-basal polarization in renal epithelial cells

**DOI:** 10.1371/journal.pcbi.1012140

**Published:** 2024-05-20

**Authors:** Maria J. Hagelaars, Milica Nikolic, Maud Vermeulen, Sylvia Dekker, Carlijn V. C. Bouten, Sandra Loerakker

**Affiliations:** 1 Eindhoven University of Technology, Department of Biomedical Engineering, Eindhoven, The Netherlands; 2 Institute for Complex Molecular Systems, Eindhoven, The Netherlands; Pázmány Péter Catholic University: Pazmany Peter Katolikus Egyetem, HUNGARY

## Abstract

Apical-basal polarization in renal epithelial cells is crucial to renal function and an important trigger for tubule formation in kidney development. Loss of polarity can induce epithelial-to-mesenchymal transition (EMT), which can lead to kidney pathologies. Understanding the relative and combined roles of the involved proteins and their interactions that govern epithelial polarity may provide insights for controlling the process of polarization via chemical or mechanical manipulations in an *in vitro* or *in vivo* setting. Here, we developed a computational framework that integrates several known interactions between integrins, Rho-GTPases Rho, Rac and Cdc42, and polarity complexes Par and Scribble, to study their mutual roles in the emergence of polarization. The modeled protein interactions were shown to induce the emergence of polarized distributions of Rho-GTPases, which in turn led to the accumulation of apical and basal polarity complexes Par and Scribble at their respective poles, effectively recapitulating polarization. Our multiparametric sensitivity analysis suggested that polarization depends foremost on the mutual inhibition between Rac and Rho. Next, we used the computational framework to investigate the role of integrins and GTPases in the generation and disruption of polarization. We found that a minimum concentration of integrins is required to catalyze the process of polarization. Furthermore, loss of polarization was found to be only inducible via complete degradation of the Rho-GTPases Rho and Cdc42, suggesting that polarization is fairly stable once it is established. Comparison of our computational predictions against data from *in vitro* experiments in which we induced EMT in renal epithelial cells while quantifying the relative Rho-GTPase levels, displayed that EMT coincides with a large reduction in the Rho-GTPase Rho. Collectively, these results demonstrate the essential roles of integrins and Rho-GTPases in the establishment and disruption of apical-basal polarity and thereby provide handles for the *in vitro* or *in vivo* regulation of polarity.

## Author summary

Apical-basal polarization of renal epithelial cells lies at the foundation of the kidney’s biological function and is the catalyst for tubulogenesis during kidney development. Although individual parts of the polarization pathway have been extensively studied before, a deep understanding of the combined and relative roles of the different proteins and/or protein interactions that lead to the emergence of polarization remains lacking due to the complexity of the various and integrated pathways. To dissect the complexity, we constructed a computational model that integrates known mechanisms of the polarization pathway. We found that the emergence of polarization is heavily dependent on a specific subset of the interactions between the involved proteins (i.e. integrins, Rho-GTPases, and polarity proteins Par and Scribble). Specifically, we concluded from our model that polarization could only be initiated when the number of integrins exceeded a threshold value, while polarization could only be disrupted in our model by complete degradation of certain Rho-GTPases. Collectively, these results indicate that apical-basal polarization is a stable process once it has emerged. Our findings help in deepening the understanding of the polarization pathway and provide new insights for regulating apical-basal polarization.

## 1. Introduction

Renal epithelial cells are responsible for the directional transport of ions, fluids, and other substances that are crucial for the kidney’s function of regulated water reabsorption and substance exchange in the renal tubules [1–3]. To enable these biological functions, the cells organize their plasma membrane and subcellular components into structurally and functionally specified subdomains by asymmetrically arranging adhesion molecules, phospholipids, protein complexes, and cytoskeletal components; a process referred to as polarization [4,5]. In this respect, apical-basal polarization refers to the process of establishing an apical membrane, facing the lumen of renal tubules, and a basolateral membrane that is in contact with neighboring cells and/or the surrounding extracellular matrix. Intracellular vectorial transport between the opposing apical and basal sides of the membrane is then enabled via differentially localized ion channels, transporters, and pumps [4], whereas the maintenance of the apical and basal subdomains is ensured by the presence of tight junctions that, together with the cortical actin cytoskeleton, create a barrier that prevents the diffusion of proteins and phospholipids between the apical and basolateral domains [5,6]. In the early stages of embryonic kidney development, apical-basal polarity triggers renal tubule formation. In this context, the formation of the apical membrane by the accumulation of apical proteins within a collective of epithelial cells marks the location where a lumen is formed *de novo* [2,7,8].

The establishment and direction of apical-basal polarity are initiated by integrin mediated signaling following cell adhesion to the matrix, a process referred to as outside-in signaling [9]. Following adhesion to the extracellular environment, integrins are able to initiate a multitude of signaling pathways of which several involve the Rho-GTPases [10]. Rho-GTPases act as molecular switches that alternate between active GTP-bound and inactive GDP-bound states. The switch between these two states is tightly regulated by guanine nucleotide exchange factors (GEFs) and GTP activating proteins (GAPs) [11]. The cross-talk between the Rho-GTPases has been suggested to regulate the localization of the apical polarity complexes Par (Par3-Par6-aPKC) and Crumbs (Crb-PALS-PATJ), and the basolateral polarity complex Scribble (Scrib-Dlg-Lgl) [11,12] to the separate apical and basal poles of the cell, either directly (by binding to the polarity proteins) or indirectly (by downstream signaling pathways).

A more complete understanding of the complex regulation and disruption of apical-basal polarity will advance our insights into kidney morphogenesis, pathologies, and control thereof. For instance, exploring the relationship between integrin formation/signaling and the subsequent signaling cascade involved in apical-basal polarization may reveal handles to control polarization, e.g. by manipulation of integrins with biomaterials, to trigger lumen formation or enhance the vectorial transport necessary for kidney function. Disruption of apical-basal polarity in adult tissues, on the other hand, can lead to the induction of epithelial-to-mesenchymal transition (EMT) [13], characteristic of many kidney pathologies. During EMT, epithelial cells gradually lose their epithelial hallmarks, including the loss of the asymmetric distribution of the polarity proteins and the destabilization of the intercellular junctions. Furthermore, the cells gain mesenchymal features, including increased motility and deposition of extracellular matrix, which lie at the basis of cancer and renal fibrosis. The increased motility, for example, is accompanied by low cellular junctions and a fast turnover of adhesion sites [14–17]. Investigating the proteins or protein interactions that govern EMT could thus provide insights into how targeting specific Rho-GTPases can affect the EMT process, to combat cancer or halt fibrosis in kidney disease.

In this study, we aimed to investigate the relative and combined roles of the different components of the polarization pathway in the emergence and loss of apical-basal polarity in renal epithelial cells, and to predict which factors are dominant throughout these processes. Since this is difficult to investigate via experiments alone, we developed a computational framework that integrates the known key elements of the polarization pathway. These include: the crosstalk between Rho-GTPases, the negative mutual feedback between the polarity complexes Par and Scribble, and the interactions between Par and the Rho-GTPases Rac and Cdc42 [10–12]. The modelling of the crosstalk between the Rho-GTPases was based on a previous model by Jilkine et al [18]. Additionally, we performed a multiparametric sensitivity analysis (MPSA) to determine which of the implemented components are the most influential in the polarization process. To date, it has been well established that outside-in signaling by integrins is an essential trigger for epithelial cells to polarize and organize properly. However, whether polarization is a switch-type process that requires a certain minimum concentration of integrins bound to the extracellular environment, or scales with the number of integrins, is still unclear. To investigate this, we computationally varied the concentration of active integrins and examined whether polarization was established in the model. Additionally, the computational framework was employed to investigate which proteins or protein interactions can disturb the polarized steady-state situation to induce EMT. To verify our results, the computational predictions were compared to data from *in vitro* experiments in which we induced EMT while quantifying GTPase levels.

## 2. Mathematical modeling

### 2.1. Computational framework of protein interactions in the polarization pathway

**Fig 1:**
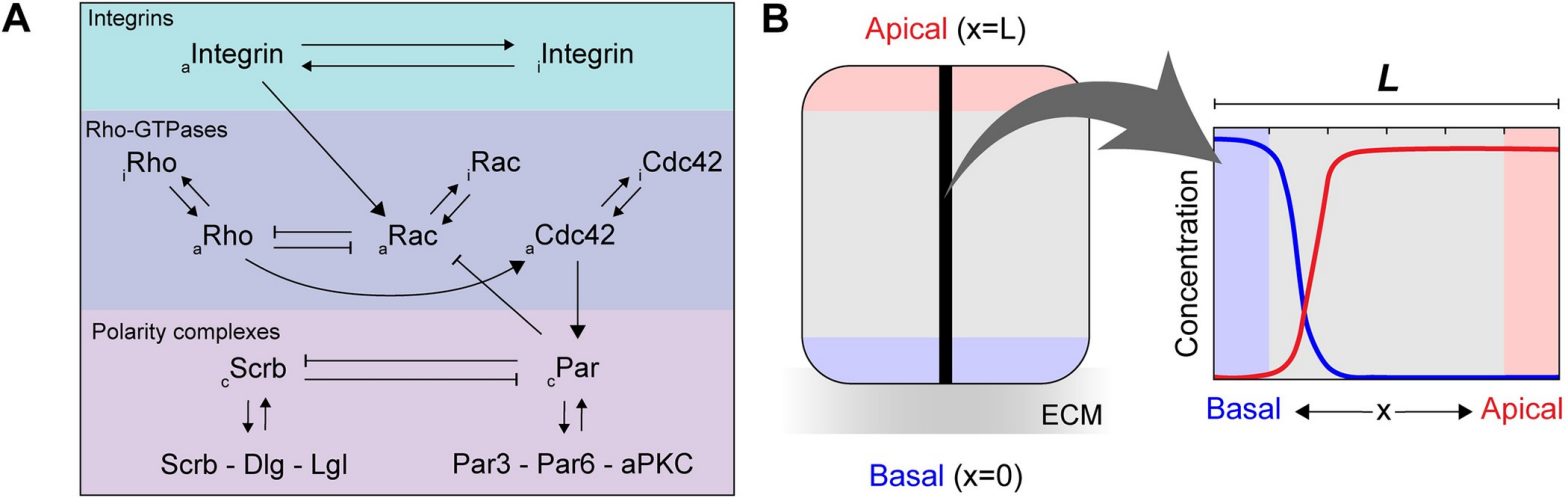
Schematic overview of the polarization model extended from [18]. (A) This scheme shows the hypothesized protein interactions that enable the establishment of polarization due to the activation of integrins. Briefly, the activation and inactivation (noted by the subscripts *a* and *i*, respectively) of integrin heterodimers (top part) influence the crosstalk between the Rho-GTPases (middle part) via upregulation of active Rac. In addition to the crosstalk between different proteins, the Rho-GTPases themselves also switch between their active an inactive forms. The bottom part displays the interaction between polarity complexes (the complex is indicated by the subscript *c*). (B) Illustration depicting examples of concentration profiles in the investigated 1D domain (0 ≤ x ≤ L*)* with the basal domain (x = 0) connected to the ECM and the apical domain (x = L) at the opposite side of the cell.

To study the emergence of polarization due to the different protein interactions, we adapted and extended the computational framework proposed by Jilkine et al [18] that describes the kinetics, crosstalk, and diffusion of the active and inactive form of the three different Rho-GTPases (Rho, Rac and Cdc42) in yeast cells. In light of our goal to understand the establishment of apical-basal polarization in renal epithelial cells, we altered the crosstalk accordingly and we added equations to model the binding kinetics, crosstalk, and diffusion of the apical polarity Par complex and the basolateral Scribble complex. Fig 1A depicts a schematic summary of the hypothesized signaling cascade responsible for the establishment of polarization in renal epithelial cells, which we implemented in our computational model. In short, the establishment of polarity is initiated by the binding of integrins to their specific extracellular matrix (ECM) ligand that causes activation of the integrins (e.g., β1 integrin) [19]. The activation of integrins then leads to the upregulation of active Rac1 [20,21]. Rac1 is in an antagonistic relation with Rho and can also be inactivated by the Par complex. Rho is responsible for Cdc42 activation that in turn upregulates the formation of the Par complex from its individual components (Par3, Par6 and aPKC). The Par complex downregulates the formation of the Scribble complex from its individual components (Scribble, Dlg, Lgl) and vice versa.

We based the antagonistic relation between Rho and Rac on the observation that the majority of the mechanisms in epithelial cells that connect Rho and Rac were found to lead to mutual inhibition [22]. The activation of Cdc42 by Rho rests on the finding that RhoGEF CGEF-1 is suggested to activate Cdc42 during the establishment of polarization [23]. We assumed that Cdc42 could stimulate the formation of the Par complex, since activated Cdc42 is known to recruit Par6 to the apical membrane [24]. The binding between Cdc42 and Par that leads to the formation of the Par-complex has been shown to be regulated by other proteins, such as the phosphoinositides [24,25]. Since these other proteins do not affect the direct relation between Cdc42 and the Par-complex, we have chosen to only model this direct relation [24,25]. Since Rho activates Cdc42 and Cdc42 in turn activates Par, a simplification in the model could be offered by modeling a direct relation between Rho and Par and thus leaving out Cdc42. We chose to omit this simplification based on the notion that Cdc42 is highly involved in the binding of Par complex and the extent of this role is not known [24,25]. As another part of the Par-complex (Par3) has been found to inhibit Tiam-Rac signaling at the apical membrane in MDCKs, we assumed that Rac is inhibited by the Par complex [12]. Finally, the downregulation of the formation of the Par-complex by the Scribble complex and the other way around is based on the known mutual inhibition of the Par and Scribble complex [26].

Collectively, the different protein interactions were modeled via a set of twelve partial differential equations (PDEs). The approximate solution of the set of PDEs was determined using the Finite Difference Method (FDM) and obtained using explicit time integration in MATLAB (R2021a, MathWorks Inc., Natick, MA, USA). The FDM is a convenient method for solving PDEs and the use of a simple 1D geometry does not require a more complex method such as the Finite Element Method. Decomposition of a set of PDEs in the FDM was performed with a forward difference in time and with a second-order central difference for the spatial derivative (as incorporated in diffusion terms). A 1D analysis along the apical-basal axis of a renal epithelial cell, giving a 1D representation of the cell, was performed on the domain 0 ≤ x ≤ L, where x = 0 and x = L denote the basal and apical boundaries of the cell, respectively (Fig 1B). Different mesh sizes were tested (10, 30 and 100 subdomains), revealing that using 30 subdomains (31 nodes) was sufficient to reach mesh convergence. Lastly, different time increments were tested (dt = 0.01, 0.005 and 0.001 seconds), showing that a time increment of 0.005 s was sufficient to ensure accuracy of the solution.

### 2.2. Model equations

The activation and inactivation of integrins are described as follows:

$$\frac{\partial I_{a}(x,t)}{\partial t}=I_{I}\frac{I_{i}(x,t)}{I_{tot}}-\delta_{I}I_{a}(x,t)$$ (1)

$$\frac{\partial I_{i}(x,t)}{\partial t}=-I_{I}\frac{I_{i}(x,t)}{I_{tot}}+\delta_{I}I_{a}(x,t)+D_{I_{i}}\frac{\partial^{2}I_{i}(x,t)}{\partial x^{2}}$$ (2)

Here, I_a_ corresponds to integrins in an activated state, while I_i_ describes the inactivated state of integrins. The activation and de-activation rates are represented by I_I_ and δ_I_ respectively. Since integrin activation is enabled by the ECM, we assume that integrin activation is only possible at the cell border connected to the ECM and that activated integrins are not able to diffuse due to their connection to the ECM. Inactive integrin on the other hand is known to be transported via vesicles, and diffusion is therefore included via the ∇^2^*I*_*i*_ term that scales with the diffusion coefficient $D_{I_{i}}$ [27]. It has to be noted though that the spatial constraint imposed on the active integrins also causes a spatial predisposition for the inactive integrins, as the total amount of integrins per element is constant and divided amongst both fractions.

The active and inactive forms of the Rho-GTPases (Cdc42, Rac and Rho) are described by Eqs 3–5 and 6–8, respectively:

$$\frac{\partial C_{a}(x,t)}{\partial t}=({\alpha_{\rho }\rho }_{a}(x,t)+I_{C})(\frac{C_{i}(x,t)}{C_{tot}})-\delta_{C}C_{a}(x,t)+D_{C_{a}}\frac{\partial^{2}C_{a}(x,t)}{\partial x^{2}}$$ (3)

$$\frac{\partial R_{a}(x,t)}{\partial t}=\frac{\left(\alpha_{I}{I_{a}(x,t)+I}_{R}\right)}{1+{\left(\frac{\rho_{a}(x,t)}{\beta_{\rho }}\right)}^{n}+{\left(\frac{P_{c}(x,t)}{\beta_{PR}}\right)}^{n}}(\frac{R_{i}(x,t)}{R_{tot}})-\delta_{R}R_{a}(x,t)+D_{R_{a}}\frac{\partial^{2}R_{a}(x,t)}{\partial x^{2}}$$ (4)

$$\frac{\partial \rho_{a}(x,t)}{\partial t}=\frac{I_{\rho }}{1+{\left(\frac{R_{a}(x,t)}{\beta_{R}}\right)}^{n}}(\frac{\rho_{i}(x,t)}{\rho_{tot}})-\delta_{\rho }\rho_{a}(x,t)+D_{\rho_{a}}\frac{\partial^{2}\rho_{a}(x,t)}{\partial x^{2}}$$ (5)

$$\frac{\partial C_{i}(x,t)}{\partial t}=-(\alpha_{\rho }\rho_{a}(x,t)+I_{C})(\frac{C_{i}(x,t)}{C_{tot}})+\delta_{C}C_{a}(x,t)+D_{C_{i}}\frac{\partial^{2}C_{i}(x,t)}{\partial x^{2}}$$ (6)

$$\frac{\partial R_{i}(x,t)}{\partial t}=-\frac{\left(\alpha_{I}{I_{a}(x,t)+I}_{R}\right)}{1+{\left(\frac{\rho_{a}(x,t)}{\beta_{\rho }}\right)}^{n}+{\left(\frac{P_{c}(x,t)}{\beta_{P}}\right)}^{n}}(\frac{R_{i}(x,t)}{R_{tot}})+\delta_{R}R_{a}(x,t)+D_{R_{i}}\frac{\partial^{2}R_{i}(x,t)}{\partial x^{2}}$$ (7)

$$\frac{\partial \rho_{i}(x,t)}{\partial t}=-\frac{I_{\rho }}{1+{\left(\frac{R_{a}(x,t)}{\beta_{R}}\right)}^{n}}(\frac{\rho_{i}(x,t)}{\rho_{tot}})+\delta_{\rho }\rho_{a}(x,t)+D_{\rho_{i}}\frac{\partial^{2}\rho_{i}(x,t)}{\partial x^{2}}$$ (8)

Here C_a_ (x,t), R_a_ (x,t), ρ_a_ (x,t) represent the concentrations of active Cdc42, Rac, and Rho at position *x* and time *t* respectively and C_i_ (x,t), R_i_ (x,t), ρ_i_ (x,t) the concentrations of inactive Cdc42, Rac, and Rho at position x and time t. C_tot_, R_tot_, ρ_tot_ represent the total concentrations for Cdc42, Rac, and Rho. The inhibition rates are indicated by δ_C_, δ_R_, δ_ρ_. The baseline activation rates of Cdc42, Rac and Rho, are represented by I_C_, I_R_, I_ρ_, respectively. β_ρ_, β_PR_ are constants that govern the rate of inhibition of Rac by the concentration of activated Rho and the concentration of the Par complex (P_C_), respectively. The inhibition is described by the Hill equation with n being the Hill coefficient. β_R_ is the constant that governs the rate of inhibition of activated Rho by the concentration of activated Rac. Inhibition and thus a decrease of activation rate is described by the Hill equation with n being the Hill coefficient. α_I_, α_ρ_ are constants that describe the activation of Rac by the concentration of active integrin and the concentration of active Rho respectively. $\frac{\partial^{2}C_{a}(x,t)}{\partial x^{2}},\frac{\partial^{2}R_{a}(x,t)}{\partial x^{2}},\frac{\partial^{2}\rho_{a}(x,t)}{\partial x^{2}}$ and $\frac{\partial^{2}C_{i}(x,t)}{\partial x^{2}},\frac{\partial^{2}R_{i}(x,t)}{\partial x^{2}},\frac{\partial^{2}\rho_{i}(x,t)}{\partial x^{2}}$ represent the second order spatial gradients that determine the diffusion of the active and inactive forms with corresponding diffusion coëfficients $D_{C_{a}},D_{R_{a}},D_{\rho_{a}}$ and $D_{C_{i}},D_{R_{i}},D_{\rho_{i}}$. We assumed that the inactive forms located in the cytosol diffuse faster than the membrane-bound active forms, which implies that $D_{C_{a}}\ll D_{C_{i}},D_{R_{a}}\ll D_{R_{i}}$ and $D_{\rho_{a}}\ll D_{\rho_{i}}$.

We based the formation and dissociation of the polarity complexes on the same principle as the switching between the active and inactive forms of the Rho-GTPases and the integrins. In this way, the total concentration of proteins is divided into two fractions: a concentration that represents the unbound proteins (Par3, Par6 and aPKC, and Scrib, Dlg and Lgl) and a concentration that represents the bound Par and Scribble complex. This leads to the following two equations for the Par complex:

$$\frac{\partial P_{C}(x,t)}{\partial t}=\frac{\left({{\alpha_{C}C}_{a}(x,t)+k}_{on,P}\right)}{1{+(\frac{S_{C}(x,t)}{\beta_{SP}})}^{n}}(\frac{P_{un}(x,t)}{P_{tot}})-k_{off,P}P_{C}(x,t)+D_{P_{C}}\frac{\partial^{2}P_{C}(x,t)}{\partial x^{2}}$$ (9)

$$\frac{\partial P_{un}(x,t)}{\partial t}=-\frac{\left({{\alpha_{C}C}_{a}(x,t)+k}_{on,P}\right)}{1{+(\frac{S_{C}(x,t)}{\beta_{SP}})}^{n}}(\frac{P_{un}(x,t)}{P_{tot}})+k_{off,P}P_{C}(x,t)+D_{P_{un}}\frac{\partial^{2}P_{un}(x,t)}{\partial x^{2}}$$ (10)

Here, P_un_ (x,t) corresponds to the collective concentration of Par3, Par6 and aPKC in an unbound state, which means that the individual values of Par3, Par6 and aPKC are not accounted for. P_C_ (x,t) describes the bound state of the Par complex. To distinguish the terms related to the polarization complexes from the terms related to the Rho-GTPases and integrins, we chose to describe the rates responsible for the switch between the bound and unbound state of the polarity complexes via k_on,P_ and k_off,P_ and refer to these as association and dissociation rates. The rate of formation of the Par complex as determined by the concentration of active Cdc42 is set by α_C_. β_SP_ governs the rate of dissociation of the Par complex as a function of the concentration of the Scribble complex. Inhibition is again described by the Hill equation with n being the Hill coefficient.

The formation and dissociation of the polarity complex Scribble is described by:

$$\frac{\partial S_{C}(x,t)}{\partial t}=\frac{k_{on,S}}{1{+(\frac{P_{C}(x,t)}{\beta_{PS}})}^{n}}(\frac{S_{un}(x,t)}{S_{tot}})-k_{off,S}S_{C}(x,t)+D_{S}\frac{\partial^{2}S_{C}(x,t)}{\partial x^{2}}$$ (11)

$$\frac{\partial S_{un}(x,t)}{\partial t}=-\frac{k_{on,S}}{1{+(\frac{P_{C}(x,t)}{\beta_{PS}})}^{n}}(\frac{S_{un}(x,t)}{S_{tot}})+k_{off,S}S_{C}(x,t)+D_{S_{un}}\frac{\partial^{2}S_{un}(x,t)}{\partial x^{2}}$$ (12)

S_un_ (x,t) represents the collective concentration for the Scrib, Dlg and Lgl in an unbound state, while S_C_ represents the bound state of the Scribble complex. The switch between the bound and unbound state corresponds to the association and dissociation rates (k_on,S_, k_off,S_). β_PS_ governs the rate of dissociation of the Scribble complex based on the concentration of the Par complex. Once more, the inhibition is governed by the Hill equation with n being the Hill coefficient.

### 2.3. Parameter estimates

Since many values of the parameters can be inferred from different cell lineages but not specifically from renal epithelial cells, we have based the majority of our parameter estimates on the composite set of data of various cell types used in Jilkine et al. [18]. Slight variations in these parameters did not lead to differences in the results of the model, indicating that the parameter values were in an acceptable range. Parameters describing the total concentrations of the Rho-GTPases were derived from own performed experiments, which are described in the experimental materials and methods section. For the parameters β_PR_ and α_ρ_, which were not included in Jilkine et al. [18], values were chosen in the same order of magnitude as the other inhibition and activation constants, for example β_R_ and α_C_ respectively. The value for I_C_ was decreased compared to Jilkine et al. [18]. The original value was suitable for the response in the GTP-ases, which is described by the original model from Jilkine et al. [18], but was not able to describe the response of the polarity complexes properly. We tested different values of I_C_ within the order of magnitude given by I_R_ and I_ρ_, and selected the value that led to larger differences in the concentration at the apical and basal pole for the polarization complexes Par and Scribble, and thus a stronger polarized distribution. The parameter values for the Rho-GTPases are summarized in Table 1.

**Table 1:** Parameter values for the Rho-GTPases in the polarization model.

Parameter	Definition	Value	Origin
C_tot_, R_tot_, ρ_tot_	Total Cdc42, Rac, and Rho concentration	4.4, 2.4, and 4.0 μM	All from experiments
I_C_, I_R_, I_ρ_	Cdc42, Rac, and Rho activation input rates	0.5, 0.5, and 3.3 μM s^-1^	Estimated, (18), (18)
δ_C_, δ_R_, δ_ρ_	Decay rates of activated Cdc42, Rac and Rho	1 s^-1^	(18)
β_R_, β_ρ_, β_PR_	Active Rac, active Rho and the Par complex dependent inhibition rate	1.5, 0.7 and 2.0 μM	(18), (18), and estimated
α_C_, α_ρ_	Cdc42 and Rho dependent activation rate	2.5 s^-1^	(18), and estimated
N	Hill coefficient of inhibition responses	4	(18)
$D_{C_{a}},D_{R_{a}},D_{\rho_{a}}$	Membrane diffusion coefficient of small GTPases	0.1 μm^2^ s^-1^	(18)
$D_{C_{i}},D_{R_{i}},D_{\rho_{i}}$	Cytosolic diffusion coefficient of small GTPases	10 μm^2^ s^-1^	(18)

**Fig 2:**
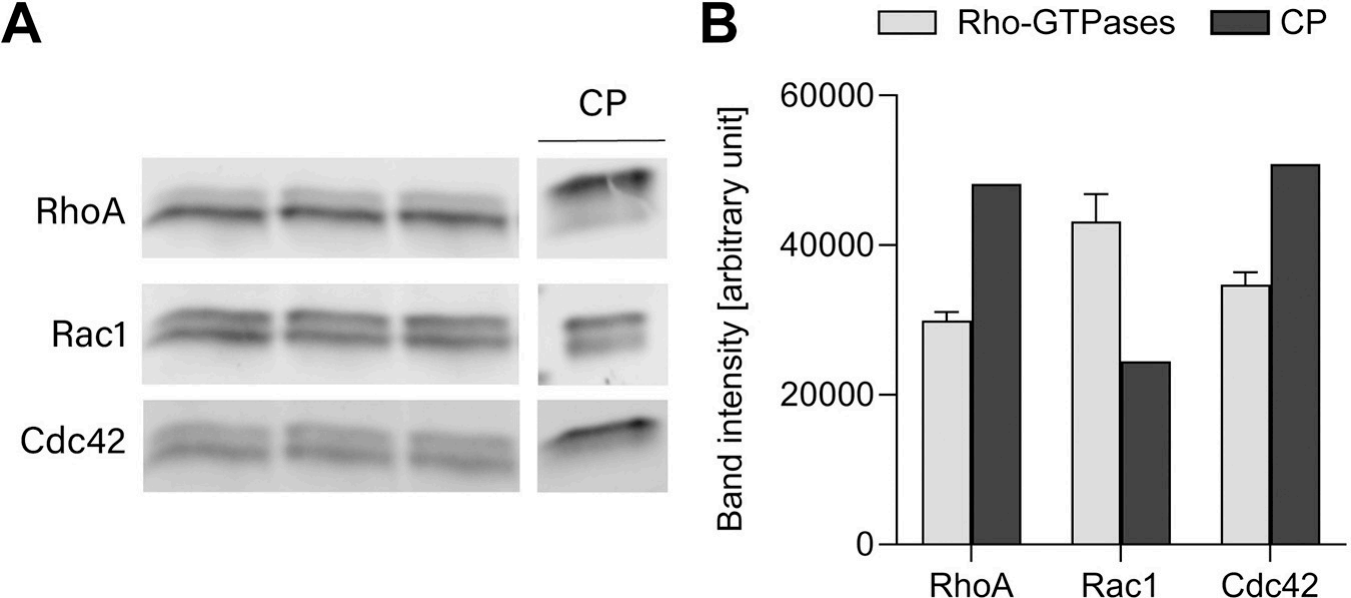
Quantification of total amount of Rho-GTPases. (A) Expression levels of RhoA, Rac1 and Cdc42 in MDCKs cultured on transwell filters and expression levels of the control protein (CP) as analyzed by western blotting using protein-specific antibodies. (B) The average band intensities for the Rho-GTPases and the band intensity of the corresponding control protein were calculated (error bars represent the standard deviation, n = 3).

In our experiments, the concentrations of small G-proteins in MDCKs were determined using immunoblotting (Fig 2A). For this, we compared the average band intensities for RhoA, Rac1 and Cdc42 in MDCKs cultured on transwell filters to the band intensity of the corresponding control protein with a known concentration (3 ng ml^-1^) (Fig 2B). Via calculations based on Marée et al. [28], we obtained the total concentrations, i.e. C_tot_, R_tot_, ρ_tot_ of 4.4, 2.4 and 4.0 μM respectively. In short, we first calculated the amount of each Rho-GTPase per lysate volume. Next, assuming that the total lysate represents 1.5 million cells, which is based on the number of MDCKs in a confluent culture, we calculated the amount of each Rho-GTPase per cell. Based on the molecular weight (21 kDa) and approximating cells as 10 μm diameter spheres, we calculated the amount of molecules per cell. With the Avogadro number we subsequently calculated the total concentrations. The total concentration of each protein was divided into an active and an inactive fraction, which were used as the initial conditions of the model. The total concentrations and initial conditions were spatially uniform and thus imposed on each node.

The parameter values for the polarity complexes and integrin activation are summarized in Table 2. The association and dissociation rates (k_on_, k_off_), and the total concentration of Par complex were taken from the work of Wen et al [29]. The diffusion rates of the Par and Scribble complex were subdivided into a cytosolic diffusion rate for the unbound proteins and a membrane diffusion rate for the entire complex [30]. We assumed that Scribble exhibits the same behavior as Par and therefore we used the same set of parameter values. Additionally, integrins are present in either an activated or an inactivated conformation. We based the total concentration of integrins on the assumption of a basal surface of 100 μm^2^ together with an integrin density of ~100 integrins per μm^2^ [31,32]. In addition, we assumed that active integrins are not able to diffuse through the cell as they are connected to the ECM, while inactive integrins are often transported via intracellular vesicles [33]. The activation and inactivation rates of these integrins were taken from previous studies and amount to 10 μM s^-1^ and 1 s^-1^ respectively [34,35].

**Table 2:** Parameter values for the polarity complexes and integrins in the polarization model.

Parameter	Definition	Value	Origin
P_tot_, S_tot_	Effective total of Par complex and Scribble complex concentration	1.6 μM	(29)
k_on,P_, k_on,S_	Par and Scribble association rate or on-rate	0.5 and 4 μM s^-1^	(29)
k_off,P_, k_off,S_	Dissociation or off-rate of bound Par and Scribble	2.0 s^-1^	(29)
β_SP_, β_PS_	Scribble-complex and Par-complex dependent inhibition rate	0.2 μM	Estimated
$D_{P_{C}},D_{S_{C}}$	Membrane diffusion coefficient of Par and Scribble complexes	0.2 μm^2^ s^-1^	(30)
$D_{P_{un}},D_{S_{un}}$	Cytosolic diffusion coefficient of unbound Par and Scribble proteins	2.0 μm^2^ s^-1^	(30)
I_tot_	Effective total of integrins	30 μM	(31,32)
I_i_	Integrin activation input rates	10 μM s^-1^	(34)
δ_I_	Decay rates of activated integrin	1 s^-1^	(35)
α_I_	Integrin-dependent activation rate	1.5 s^-1^	Estimated
$D_{I_{i}}$	Cytosolic diffusion coefficient of inactive integrins	10 μm^2^ s^-1^	(30)

### 2.4. Initial and boundary conditions

Since we considered that active integrins exist only close to the membrane, initial values of integrins were assigned to the first five nodes (one-sixth of the domain and thus approximately 1.67 μm) with an exponential decay (Eq 13):

$$I_{a}(x,0)=\frac{1}{x^{2}}∙e^{\left(\frac{1}{x^{2}}-1\right)}∙I_{tot}$$ (13)

Here, x ranges from 1 to 5, representing the first five nodes and thus 1.67 μm starting from the basal side. Homogeneously distributed initial concentrations of active Rho-GTPases and Par and Scribble complexes were applied with C_a_ (x,0) = 0.8 μM; ρ_a_ (x,0) = 1.0 μM; R_a_ (x,0) = 1.75 μM and P_C_ (x,0) = S_C_ (x,0) = 0.6 μM. Concentrations of the initial inactive Rho-GTPases and unbound polarity complexes were defined as the difference between the conserved total concentration and the active concentration of the protein in question. The initial spatial uniform input values for the active and inactive fractions were changed compared to Jilkine et al [18]. (C_a_ (x,0) = 1.00 μM; ρ_a_ (x,0) = 1.25 μM; R_a_ (x,0) = 3.00 μM), as we aimed to model renal epithelial cells instead of yeast cells. We chose the overall fraction in the GTP-bound-state at steady state to be around 30%, based on the comparison to erythrocytes and neutrophils [36,37]. We assumed that the percentage of activated Rac1 and Cdc42 is two times higher than the percentage of activated Rho (30% compared to 15%), in correspondence to previously experiments involved with other epithelial cells [38–40].

At the outer cell membrane, a no-flux boundary condition was prescribed for all diffusible substances: $\frac{\partial }{\partial x}$ C = 0 at x = 0, L. Boundary conditions were imposed as the first order backward differences at the basal side and as the first order forward differences at the apical side.

### 2.5. Multiparametric sensitivity analysis (MPSA)

Our computational framework includes a large number of parameters, of which some have a high level of uncertainty. This specifically concerns the parameters that define interactions between different proteins, as the strength of their interactions (e.g., the strength of inbition of Rac on Rho) and significance of the individual interactions in the polarization pathway are not known. Therefore, we performed a multiparametric sensitivity analysis (MPSA) to investigate which of our parameters have the strongest influence on establishing apical-basal polarization. To this end, we selected a set of 21 parameters (I_c_, δ_c_, α_c_, I_R_, δ_R_, β_R_, I_ρ_, δ_ρ_, α_ρ_, β_ρ_, k_on,P_, k_off,P_, β_PR_, β_PS_ k_on,S_, k_off,S_, β_SP_, I_I_, δ_I_, I_tot_, α_I_ ∈ X_V_) from the total set of 38 parameters. These parameters were chosen, as they are essential constants in the equations that describe the interactions between different proteins that cannot be measured directly in experiments.

We randomly changed the 21 parameters (X_V_) simultaneously and uniformly over the whole domain in ranges of 5 times lower to 5 times higher values (20–500% of the original parameter values displayed in Tables 1 and 2). High ranges for variation were chosen, as most of the parameters could not be determined experimentally or derived from previous studies. We varied these 21 parameters in the predefined range (20–500% of the original parameter value) using Latin hypercube sampling to create 3000 samples of parameters space [41–43]. A Monte Carlo sampling scheme was used to select 3000 parameter combinations to run simulations in parallel and assess the obtained output results in relation to the prescribed threshold. The simulation with the original parameter values (displayed in Tables 1 and 2) served as the reference case (X_O_).

In each of the 3000 simulations, we calculated six outputs (Y) from the model: the concentrations of active integrin, Rac, Rho and Cdc42, and the concentrations of the Par-complex and Scribble-complex (I_a_, R_a_, ρ_a_, C_a_, P_C_, S_C_ ∈ Y). For each output (n = 1, 2, …, 6) and each simulation (m = 1, 2, …, 3000), we determined the absolute difference between the concentration at the apical (${Y_{A}}_{m,n}$) and basal (${Y_{B}}_{m,n}$) side for a given set of the 21 parameters (${X_{V}}_{m}$) relative to the original absolute difference (X_O_) in percentages:

$$\Delta P_{m,n}=\frac{\left|{Y_{A}}_{m,n}({X_{V}}_{m})-{Y_{B}}_{m,n}({X_{V}}_{m})\right|}{\left|{Y_{A}}_{m,n}(X_{O})-{Y_{B}}_{m,n}(X_{O})\right|}∙100$$ (14)

We defined that the system achieved polarization when Δ*P* had a value higher than 20%. Therefore, we assigned value 1 to simulations with ΔP > 20% (‘accepted’) and value 0 to simulations with ΔP ≤ 20% (‘unaccepted’).

**Fig 3:**
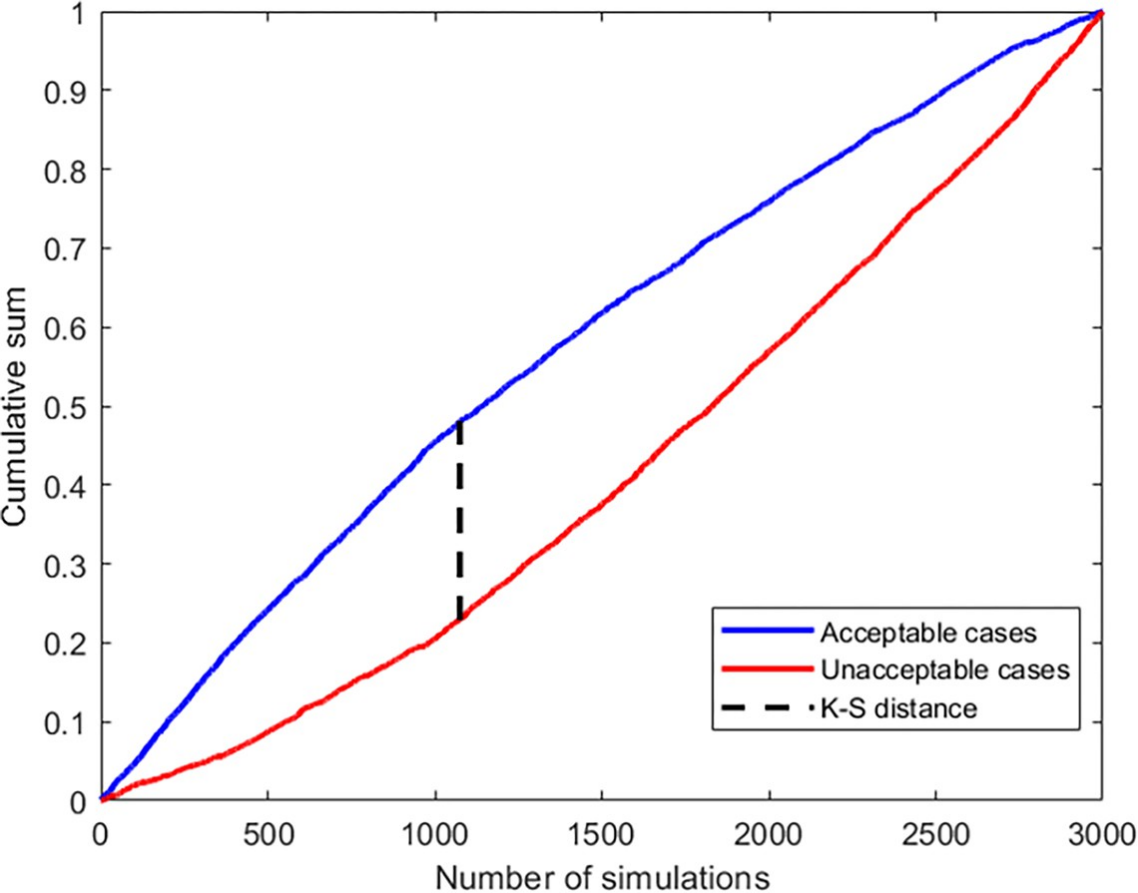
Example of the maximal absolute distance or K-S distance between the acceptable and unacceptable distribution. The example portrays the K-S distance for the parameter *β*_*R*_ with respect to Rho output. The K-S distance is defined as the maximal absolute distance between the distribution of acceptable and unacceptable cases.

The influence of each parameter (p_i_ ∈ X_V_) on the six outputs (Y) was then statistically evaluated using the Kolmogorov-Smirnov (K-S) test [44]. To perform the statistical test for each of the six outputs (Y), we considered the 21 parameters one by one. We assigned arrays of 0 and 1’s (unacceptable vs acceptable simulations) to each value of the parameter. Subsequently, the values of each parameter were sorted and cumulative distributions for the unacceptable and acceptable cases were created. For each parameter (p_i_ ∈ X_V_), the unacceptable and acceptable cumulative distributions were normalized to the corresponding maximum values of the respective cumulative distributions. Finally, the absolute distances between the acceptable and unacceptable distribution values for each of the parameter values were calculated. An example of the distance between the acceptable and unacceptable distributions is given in Fig 3. The maximal absolute distance for each parameter and each output is then the K-S distance and is represented by:

$$d_{K-S}{\left(p_{i}\right)}_{n}={max}_{n}|S_{a}(p_{i}(n))-S_{u}(p_{i}(n))|$$ (15)

where S_a_ and S_u_ are the cumulative distributions of acceptable and unacceptable cases, respectively, n stands for the output serial number (n = 1, 2, 3, …, 6) and p_i_ represents one parameter within the set of 21 parameters X_V_. Thus, for each output the influence of each of the 21 parameters is explained by the K-S distance, where larger values indicate increased influence compared to smaller values.

Together with the 21 input parameters, which were selected out of total 38 input parameters (listed in Tables 1 and 2) to be subjected to the sensitivity analysis, we included an additional set of 21 random values; the so called ‘dummy’ values. With these values, we calculated cumulative distributions and K-S distances in the same manner as for the 21 input parameters to determine the threshold sensitivity criteria. Dummy values were set at a value of 1 and were varied in the same range (20–500%) as the 21 parameters, so we obtained 3000 sets of dummy values as well. Subsequently, we assigned the same arrays of 0 and 1’s (unacceptable vs acceptable simulations) to each value of the dummy values as we did for the parameters and calculated the K-S distance for each dummy value using the same method. From the final 21 distances obtained from the dummy values and for each output, the maximal distance is taken to represent the threshold for the parameters’ sensitivity. Thus, K-S distances of the parameters that are larger then the maximal K-S distance of all dummy values considered to have a significant influence on the model output.

### 2.6 Potential impact of the degradation or upregulation of Rho-GTPases on the induction of EMT

**Fig 4:**
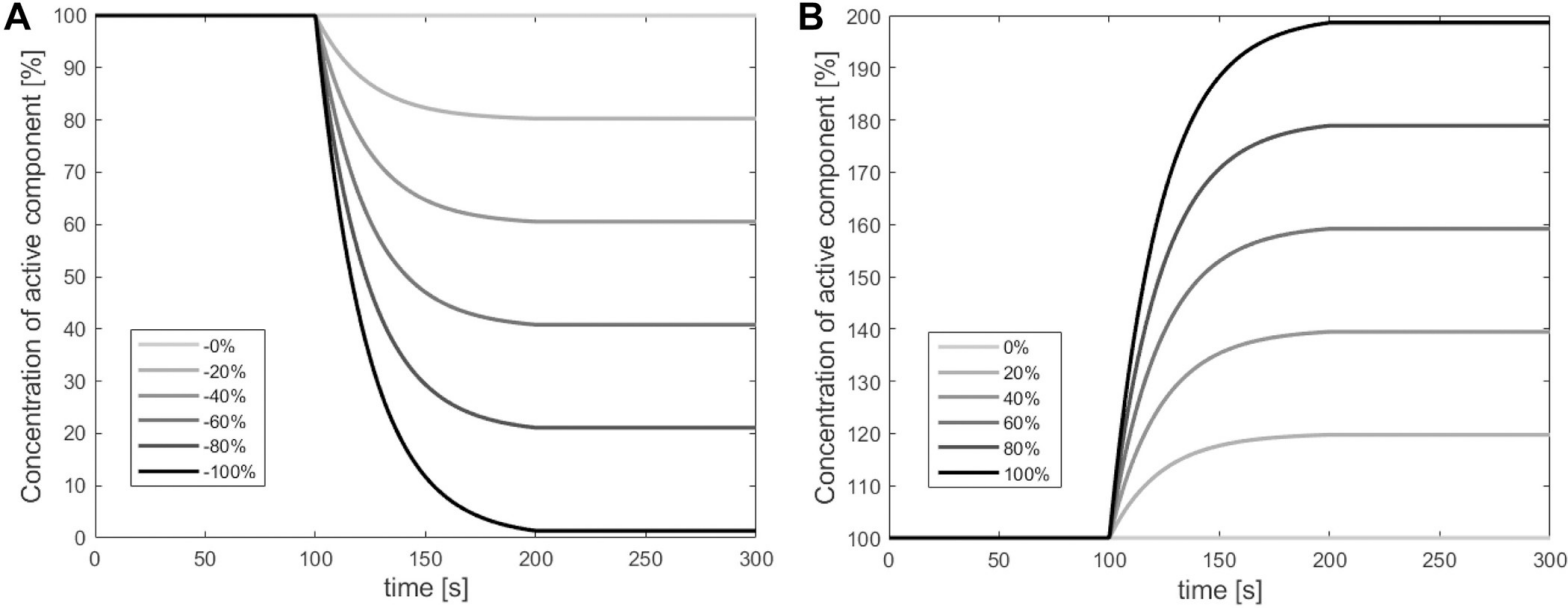
Different temporal profiles were prescribed to describe the degradation and upregulation of each of the Rho-GTPases. The temporal profiles ranged between no (0%) to full (-100%) degradation (A) and no (0%) to full (100%) upregulation (B) of the active component of each of the Rho-GTPases.

The importance of the Rho-GTPases for establishing polarization was simulated by individually degrading or upregulating the active part of each Rho-GTPase (C_a_, R_a_, ρ_a_) and updating the total amount of proteins accordingly. Exponential functions were used to capture the behavior of degradation observed in the experimental results of Abi Habib et al. [45], who investigated the degradation patterns of ubiquitinated proteins by different proteasomes. Similar exponential curves were employed to model increases of the active part of each Rho-GTPase over time. Different degrees of degradation or upregulation were imposed, ranging from 0% to ±100% of the protein in question (Fig 4). The general temporal profiles for degradation and upregulation are given by:

$$P(t)=\left\{ \begin{array}{c} 100,0\leq t\leq 100\\ 100+a{(1-e}^{-0.043(t-100)}),100<t\leq 200\\ P(t=200),200<t\leq 300 \end{array}\right.$$ (16)

With P representing the percentage of the active Rho-GTPase (C_a_, R_a_, ρ_a_) over time (t) in seconds, and a = -100, -80, …, 80, 100 the percentage of degradation (a < 0) or upregulation (a > 0) in steps of 20%. All degradation and upregulation simulations were performed by first running the simulations with the original concentrations of the Rho-GTPases for 100 seconds to achieve a polarized steady state for all proteins. After these 100 seconds, the degradation or upregulation of one of the Rho-GTPases was prescribed for a period of 100 seconds. In this period, the degradation/upregulation of the active part of the selected Rho-GTPase, together with updating the total amount of the selected Rho-GTPase, was performed at the end of each time step. The inactive parts were then updated according to Eqs 3–8. After the degradation or upregulation period, the concentrations of the degraded or upregulated Rho-GTPases were kept constant for an additional 100 seconds.

## 3. Results

### 3.1. The proposed computational framework recapitulates key features of apical-basal polarization in renal epithelial cells

**Fig 5:**
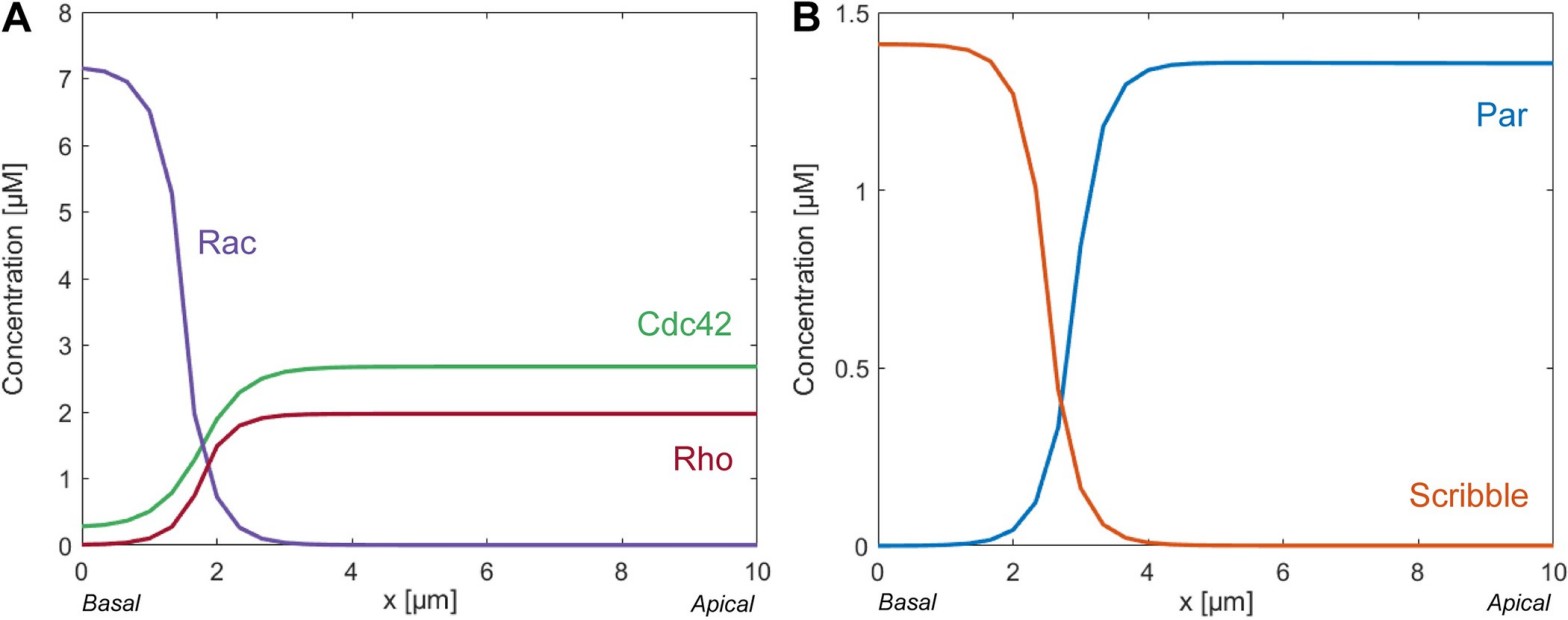
The hypothesized interplay between intracellular proteins, resulting from the localization of active integrins at the basal membrane, recapitulates the establishment of polarization in renal epithelial cells. Final steady-state distribution of the active forms of the Rho-GTPases in the model (A) and of the formed polarity complexes Par and Scribble (B). Total simulation time is 100 seconds.

To simulate apical-basal polarization, we developed a computational framework that describes the interactions between the different proteins hypothesized to be involved in the polarization pathway. The model predicted the distributions of the active forms of the Rho-GTPases and the distributions of the formed polarity complexes upon reaching equilibrium. The crosstalk included in our model, together with the chosen parameter values, caused the distribution of the active forms of the Rho-GTPases to evolve toward a steady-state polarized profile (Fig 5A). Active Rac accumulated at the basal membrane due to its activation by active integrins situated at the basal membrane. The sideways inhibition between Rac and Rho caused the active form of Rho to accumulate at the apical pole of the cell. Cdc42 colocalized with Rho because the activation of Cdc42 is dependent on Rho.

The polarized profile of the Rho-GTPases subsequently led to a steady-state polarized profile for the polarity complexes Par and Scribble in our model (Fig 5B). This concurs with the establishment of apical-basal polarization in renal epithelial cells, where the Par-complex accumulates at the apical domain and the Scribble-complex accumulates at the basal domain. The localization of Par at the apical membrane is caused by the fact that Cdc42 positively contributes to the formation of the Par complex. The transition point in the distribution of the concentrations of the Rho-GTPases and therefore also of the polarity complexes was centered closer to the basal domain of the cell. This is due to the strong dependence of our model on the distribution of active integrins for the induction of Rac activation. In the proposed computational model, these active integrins were assumed to be present only within the basal region that represents one sixth of the domain. This restriction to the basal region can be explained by our assumpiton that integrin activation is enabled by the ECM at the basal side and that they do not diffuse as they are connected to the ECM.

**Fig 6:**
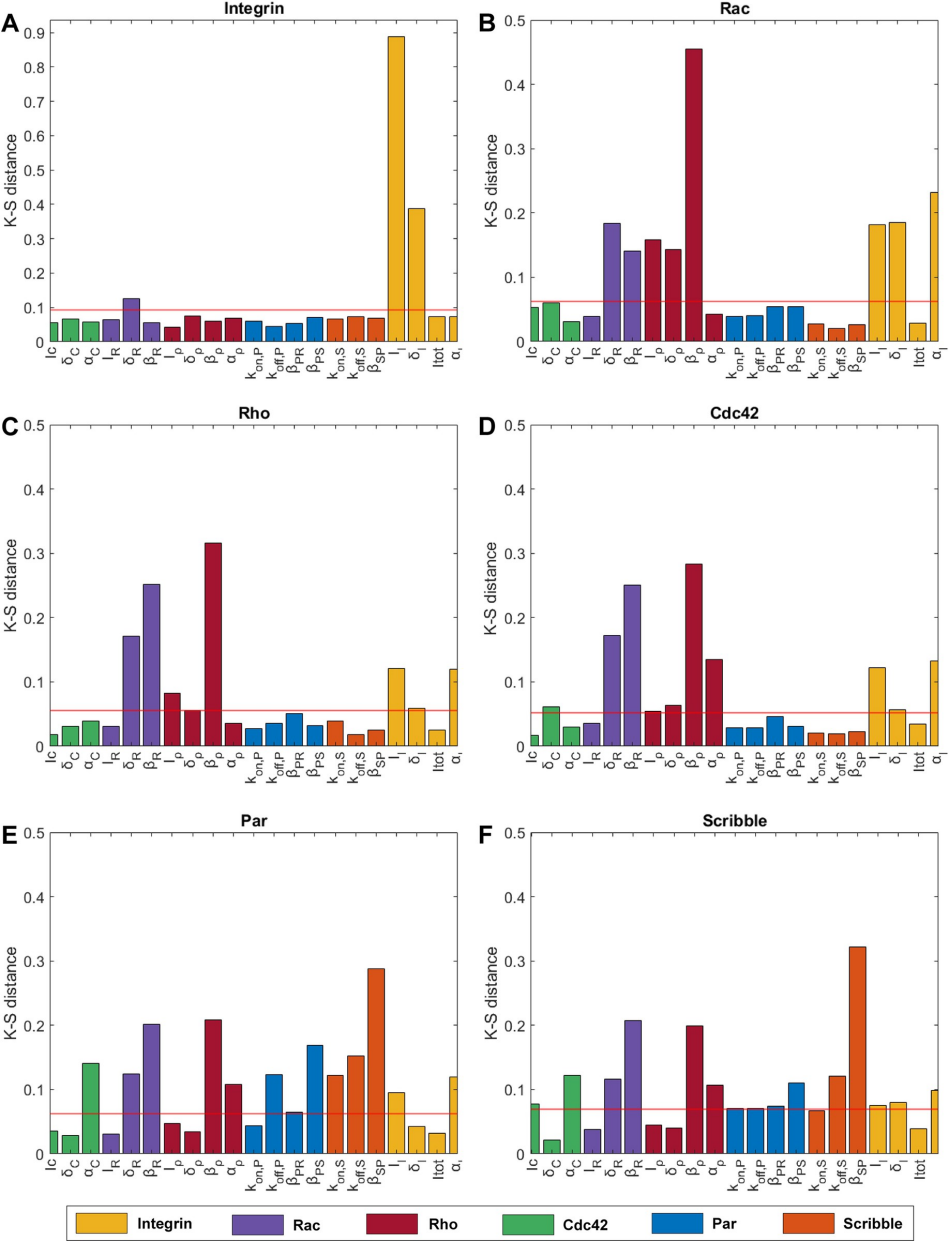
Sensitivity of polarized profiles for the six output variables to model parameters. All subfigures show the Kolmogorov-Smirnov distances (K-S distances) normalized to the maximum distance. We show the influence of 21 parameters on the polarized distributions developed for (A) activated integrin, (B) Rac, (C) Rho, (D), Cdc42, (E), Par and (F) Scribble. These 21 parameters have been grouped in color to display which of the parameters belongs to a certain protein. The horizontal red line represents the maximal K-S distance obtained from the set of dummy values. The parameters with a increased K-S distance compared to the maximal K-S distance obtained from the dummy values were considered to be influential.

Next, we aimed at elucidating which factors in our model have the largest impact on the establishment of polarization in renal epithelial cells using the MPSA. Achieving a polarized distribution for activated integrins was observed to depend completely on the parameters involved in the switch between active and inactive integrins (I_I_, δ_I_) (Fig 6A), which was expected since integrins are not connected to the rest of the pathway in our model. The establishment of polarized steady-states for the Rho-GTPases were all found to be heavily influenced by the parameters that belonged to Rho and Rac (i.e., δ_R_, β_R_, I_ρ_, δ_ρ_, α_ρ_, β_ρ_) (Fig 6B–6D). Especially the inhibition parameters β_ρ_ and β_R_, which define the inhibition rates for Rho and Rac as a function of Rac and Rho respectively, displayed very high values for the K-S distance, demonstrating their relevance. Additionally, we observed that the output of the Rho-GTPases was primarily influenced by the parameters affiliated with the integrins (I_I_, δ_I_, α_I_), with the output of Rac being the most affected by these parameters (Fig 6B). Obtaining a polarized output for both polarity complexes was found to be the most sensitive to the inhibition parameter β_SP_ that governs the rate of dissociation of the Par complex as a function of the concentration of the Scribble complex (Fig 6E and 6F). Additionally, the polarization of the polarity complexes was sensitive to changes in all other parameters, which can be explained by the dependence of the distribution of Par and Scribble on the crosstalk between the different Rho-GTPases and by the sideways inhibition of Par and Scribble. Based on the general observation that the parameters that belonging to Rho and Rac heavily affected all outputs, we conclude that the establishment of polarization in our computational framework is mainly dependent on the strength of the mutual inhibition of Rho and Rac.

**Fig 7:**
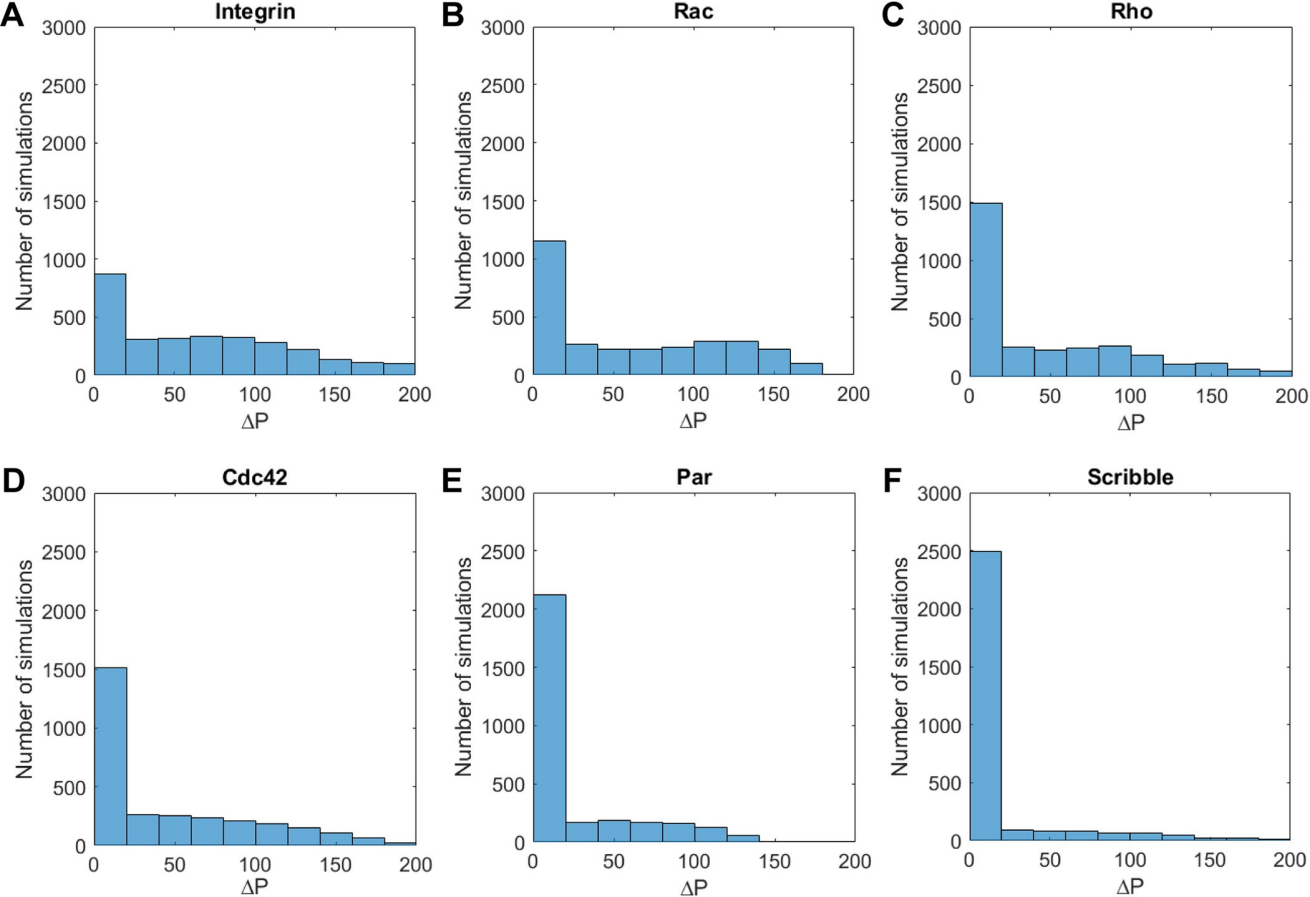
Establishing a polarized profile for the six output variables is not equally sensitive to the variation of parameters. All subfigures display the frequency of the various relative changes in concentration difference between the apical and basal side. Output sensitivity to the 21 parameters is shown for (A) activated integrin, (B) Rac, (C) Rho, (D) Cdc42, (E) Par, and (F) Scribble. The first bar (0<Δ*P*≤20%) represents the number of simulations in which polarization was not achieved.

In addition to determining the most influential parameter(s), we also analyzed which one of the six outputs is most sensitive to the changes in parameters. To this end, we inspected the ΔP for all the 3000 simulations per output variable (Fig 7). The sensitivity of the output was then related to the number of simulations from the total of 3000 simulations that resulted in no polarization or only weak polarization (ΔP < 20%). In case of a high number of simulations resulting in no polarization or weak polarization, the output was considered to be sensitive to the parameters. We found that the establishment of a polarized output for active integrins depends the least on changes of our parameters, since the output has the lowest number of simulations with a ΔP lower than 20% (Fig 7A). The notion that active integrins is the least sensitive output is again as expected since integrins are not connected to the rest of the pathway. Achieving a polarized state for Scribble was determined to be the most dependent on the parameters (Fig 7E). This observation can be explained by the fact that Scribble is at the end of the pathway where its concentration is determined by the interplay of all other proteins.

### 3.2. The establishment of apical-basal polarization requires a minimum level of activated integrins

Previous studies have demonstrated that integrins play a crucial role in the establishment of polarization, as inhibition of different integrins led to the prevention of cyst polarization in 3D environments [46–48]. Motivated by this, we investigated how interactions between integrins and the Rho-GTPases could affect the polarized profile of the polarity complexes Par and Scribble. First, we confirmed that active integrins provide the direction of the polarization response. The active integrins interacted with Rac at one side of the cell, which triggered the subsequent interactions between the Rho-GTPases, and subsequently caused the apical membrane as marked by Par to emerge at the opposite end of the cell in our model (S1 Fig). We then investigated whether the concentration of active integrins affects the establishment of a polarized profile for Par and Scribble.

**Fig 8:**
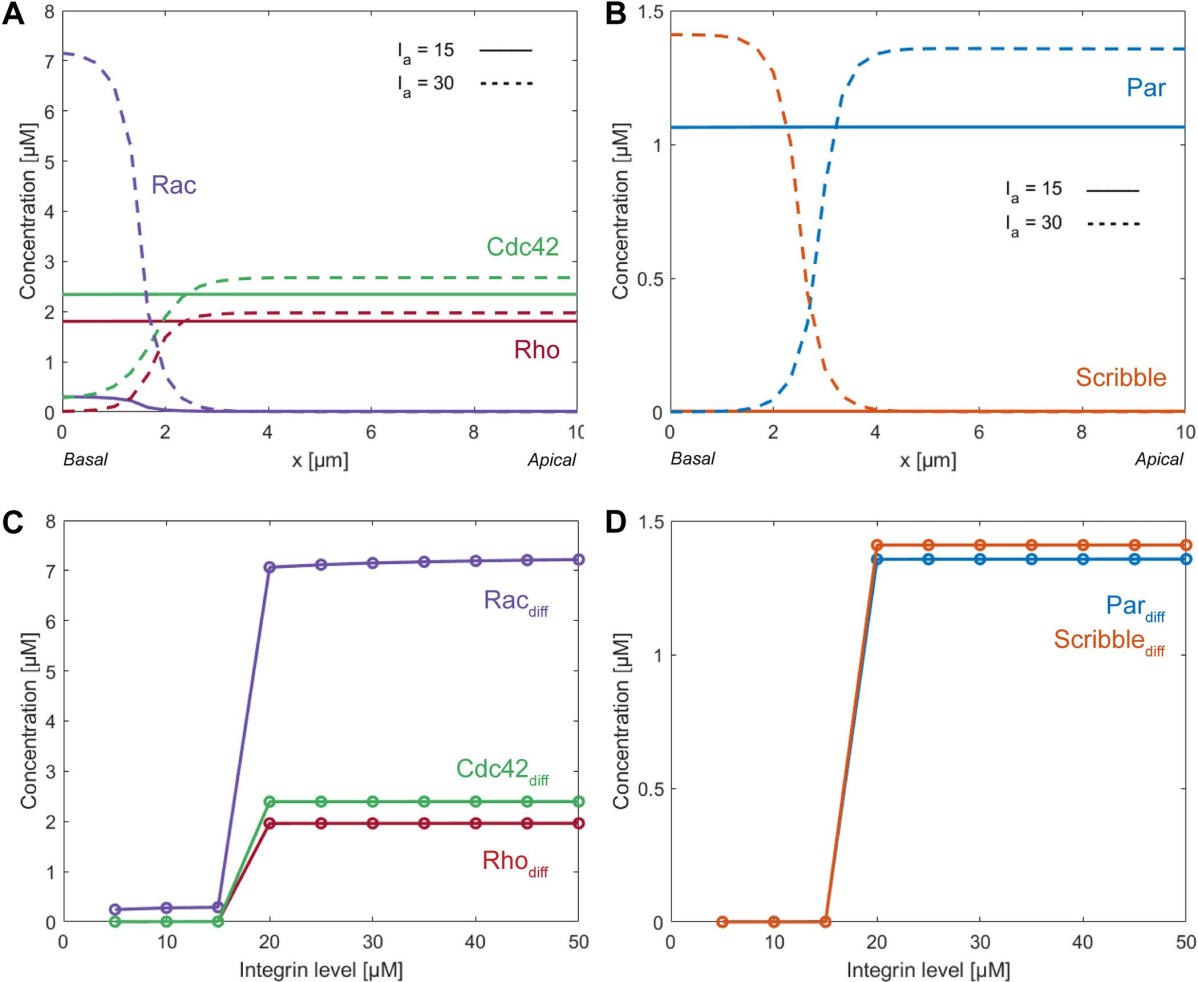
The concentration of active integrins affects the distribution of Rho-GTPases and the emergence of polarization. Examples of the final steady-state distribution of the active forms of the Rho-GTPases (A) and the formed polarity complexes Par and Scribble (B) in case of different concentration of integrins. The absolute differences in concentration of the Rho-GTPases (C) and the polarity complexes (D) between the apical and basal side show that a minimum value of 18 μM active integrins is necessary to enable the emergence of polarization.

We found that when the initial concentration of active integrin was kept below 18 μM, the active integrins were not able to generate sufficient Rac activation to induce the development of a polarized profile for the Rho-GTPases (Fig 8A). As a result, the polarity complexes Par and Scribble also remained homogeneously distributed over the cell (Fig 8B). On the other hand, initial concentrations of active integrin exceeding a threshold of 18 μM activated Rac to the degree that it caused a sufficient amount of Rho and Cdc42 to accumulate at the opposite side of the cell, thereby leading to induced polarized distributions of the Rho-GTPases (Fig 8A). The accumulation of Rho and Cdc42 at the apical side of the cells is necessary to achieve the polarized distribution for the polarity complexes Par and Scribble (Fig 8B). The strength of polarization for the Rho-GTPases and the polarity complexes, which is defined as the absolute difference in concentration between the apical and basal side, was found to be independent of the active integrin activation as long as this exceeds 18 μM (Fig 8C and 8D). Initial concentrations of active integrins higher than the threshold value of 18 μM led to only slightly higher values for Rac concentrations (Fig 8C). This stabilization of the Rac concentrations can be explained by the fact that the active concentration of Rac can only reach a maximum value given by the constant total Rac concentration. These small differences in Rac concentration however do not translate into differences in the polarized distributions of Rho and Cdc42 (Fig 8C), and therefore the distributions of the polarity complexes also remain unaffected (Fig 8D). Collectively, these results imply that the establishment of polarization in renal epithelial cells requires the concentration of active integrins to exceed a certain threshold value, which in our model corresponds to 18 μM, while the strength of polarization is independent of the concentration of active integrins beyond this threshold.

### 3.3. Full degradation of Rho leads to a loss of polarity

During the process of EMT, epithelial cells gradually lose their tight junctions, apical-basal polarization, and cell height, while they produce more actin fibers and specifically α-smooth muscle actin (α-SMA) and obtain a spreading morphology. Although the transforming growth factor (TGF) β1 is one of the most well-known inducers of EMT in renal epithelial cells, the signaling pathways by which TGF-β1 induces EMT are less well understood [49–51]. One of the hypotheses is that TGF-β1 causes the degradation of the Rho-GTPases, subsequently activating further signaling cascades that cause the induction of EMT [52,53]. In contrast, other studies have suggested that TGF-β1 can cause an induction of EMT by upregulating the Rho-GTPases [54,55]. To explore the validity of these hypotheses and to examine which TGF-β1 mediated changes in Rho-GTPases could explain EMT, we investigate whether degradation or upregulation of certain Rho-GTPases could induce a loss of polarity within our computational framework. To this end, degradation and upregulation ranging from no (0%) to a full (100%) degradation or upregulation of each Rho-GTPase was imposed. Next to this, we performed *in vitro* experiments in which we induced EMT in renal epithelial cells and subsequently measured GTPase levels relative to control conditions. The changes in these relative GTPase levels were then compared to the variations in the GTPase distributions computed in our model to examine which of the three Rho-GTPases could play a role in the TGF-β1 induced EMT.

**Fig 9:**
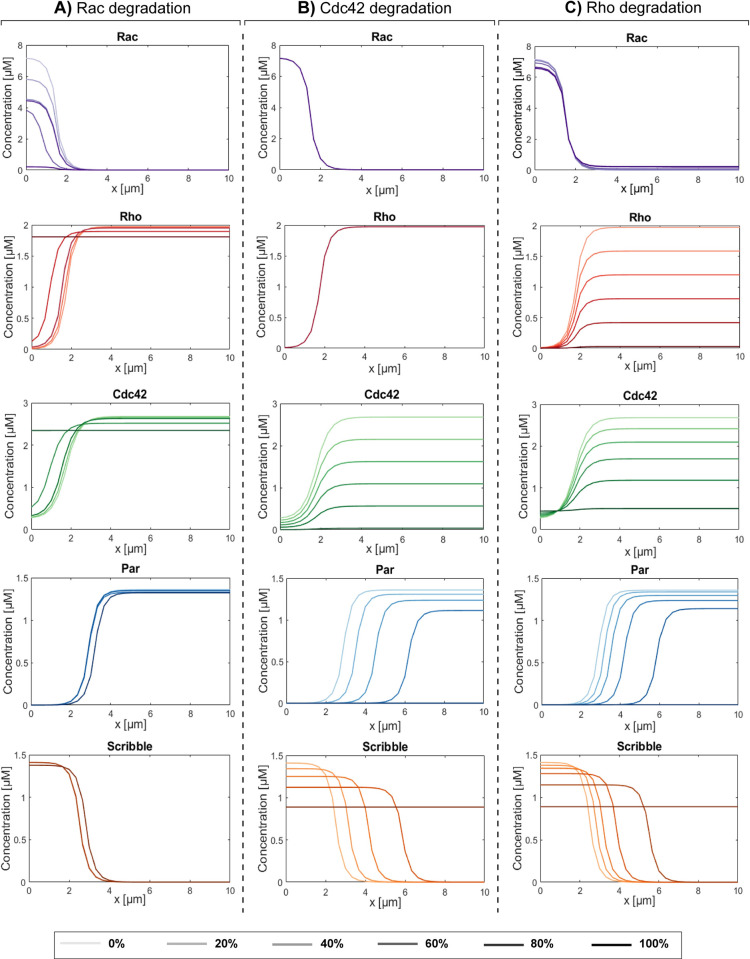
Full degradation of Rho and Cdc42 can both lead to loss of polarity in the model. Final steady-state distributions of the active forms of the Rho-GTPases and of the formed polarity complexes Par and Scribble after imposing Rac degradation (A), Cdc42 degradation (B) and Rho degradation (C). Total simulation time is 300 seconds.

First, degradation of each of the Rho-GTPases was simulated. Partial degradation (up to 80%) of active Rac, Rho, or Cdc42 was not able to induce a loss of polarization in our model, because the other Rho-GTPases and the polarity complexes preserved their polarized distributions upon partial degradation of individual Rho-GTPases (Fig 9). Complete degradation of Rac, however, led to homogeneous distributions of Rho and Cdc42 (Fig 9A), as a certain minimum concentration of Rac at the basal side is necessary for Rho and Cdc42 to accumulate at the apical side. Nevertheless, although Rho and Cdc42 lose their polarized distributions, the concentration of Cdc42 at the apical side remains at a sufficiently high level to prevent the dissocation of the Par complex, thereby preserving the polarized distributions of both polarity complexes. On the other hand, complete degradation of Cdc42 did not affect the distributions of Rac and Rho, which was expected since Cdc42 does not influence Rac or Rho in the proposed crosstalk. The complete degradation did cause the dissociation of the Par complex because a certain concentration of Cdc42 is required to maintain the Par complex (Fig 9B). Due to the mutual inhibition between Par and Scribble, the dissociation of the Par complex throughout the cell immediately resulted in a homogeneous distribution of the Scribble complex, thus causing a loss of polarization. Finally, complete degradation of Rho was shown to lead to a homogeneous distribution and lower concentrations of Cdc42, while the distribution of Rac was hardly affected (Fig 9C). The combination of fully degraded Rho and lower, homogeneously distributed concentrations of Cdc42, in turn, caused the dissociation of the Par complex. In case of Rho degradation, Cdc42 is still present in lower concentrations and may therefore not entirely explain the dissociation of the Par complex. The Scribble complex may also be involved in the dissociation of the Par complex, due to its inhibitory effect on the formation of the Par complex. Collectively, our model indicates that full degradation of either Rho and/or Cdc42 can lead to a loss of polarity during the initiation of EMT.

**Fig 10:**
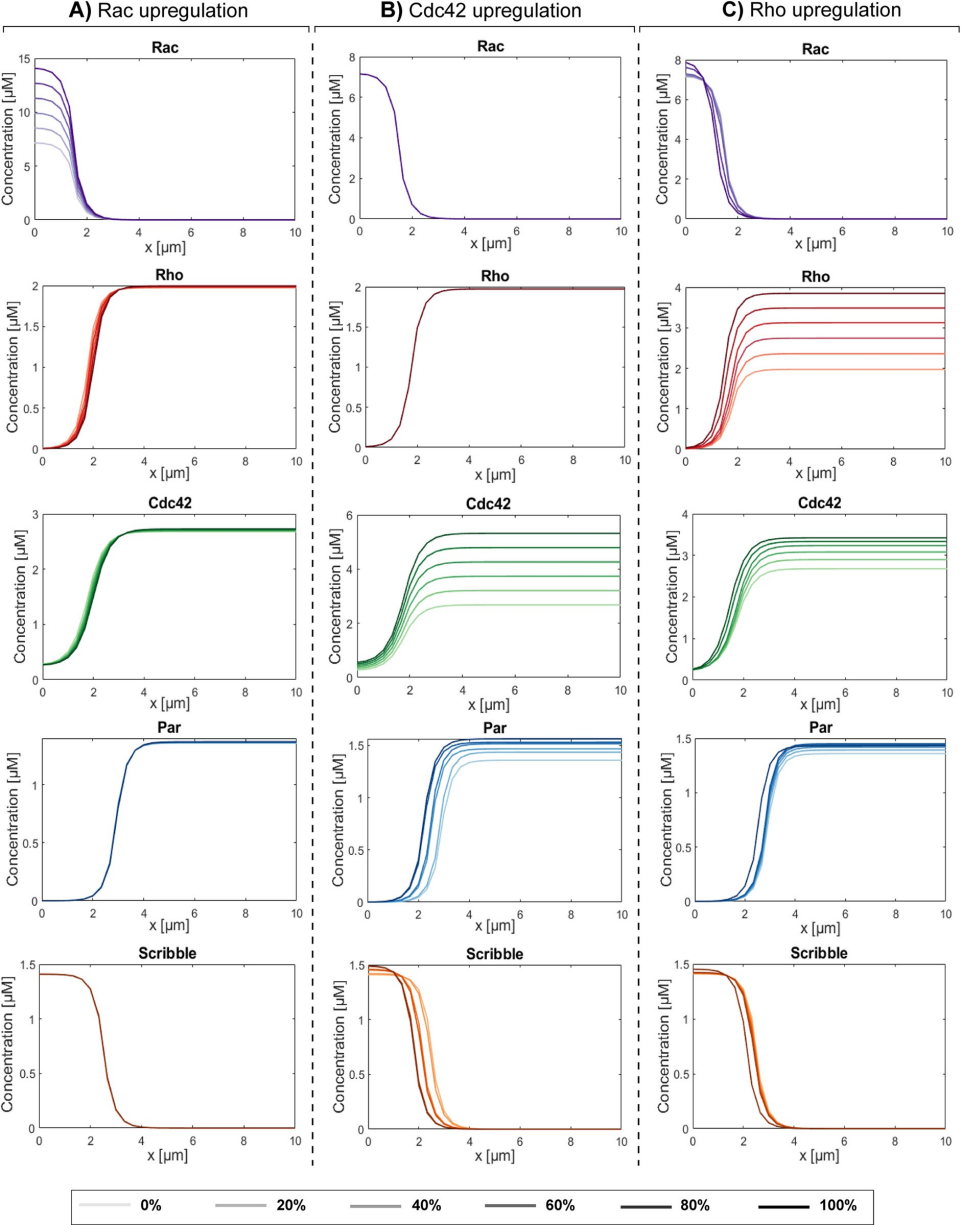
Upregulation of the Rho-GTPases does not lead to a loss in polarity. Final steady-state distributions of the active forms of the Rho-GTPases and of the formed polarity complexes Par and Scribble after imposing Rac upregulation (A), Cdc42 upregulation (B) and Rho upregulation (C). Total simulation time is 300 seconds.

Next, upregulation of each of the Rho-GTPases was simulated. In all cases, upregulation of the active part of the Rho-GTPases did not lead to a loss of polarized distribution of the Rho-GTPases as well as the polarity proteins Par and Scribble (Fig 10). Upregulation of Rac was shown to have no influence on the polarized distributions of all other proteins (Fig 10A). Our previous results (section 3.1) already showed that a certain minimum amount of Rac is necessary to allow for the accumulation of sufficient Rho and Cdc42 at the apical side to achieve and preserve polarization. The Rac concentration does not affect the concentrations of Rho and Cdc42 beyond this threshold value, as each GTPase satisfies mass conservation and thus the maximum amount of the active part cannot exceed the total amount in the cell. An upregulation of Cdc42 on the other hand slightly enhances the polarized distributions of the polarity proteins (Fig 10B) as a higher concentration of Cdc42 would in turn lead to an increase in the formation of the Par complex at the apical side of the cell and therefore also to the formation of the Scribble complex at the basal side of the cell. Lastly, an upregulation of Rho also leads to a slight enhancement in the polarized distributions of the polarity proteins (Fig 10C). This can be explained by the fact that an upregulation in Rho also leads to an upregulation in Cdc42, which in turn again causes a rise in the amount of the polarity proteins at the different poles of the cell. Together, our model indicates that upregulating the Rho-GTPases does not lead to a loss of polarity and can even slightly enhance the polarized state.

**Fig 11:**
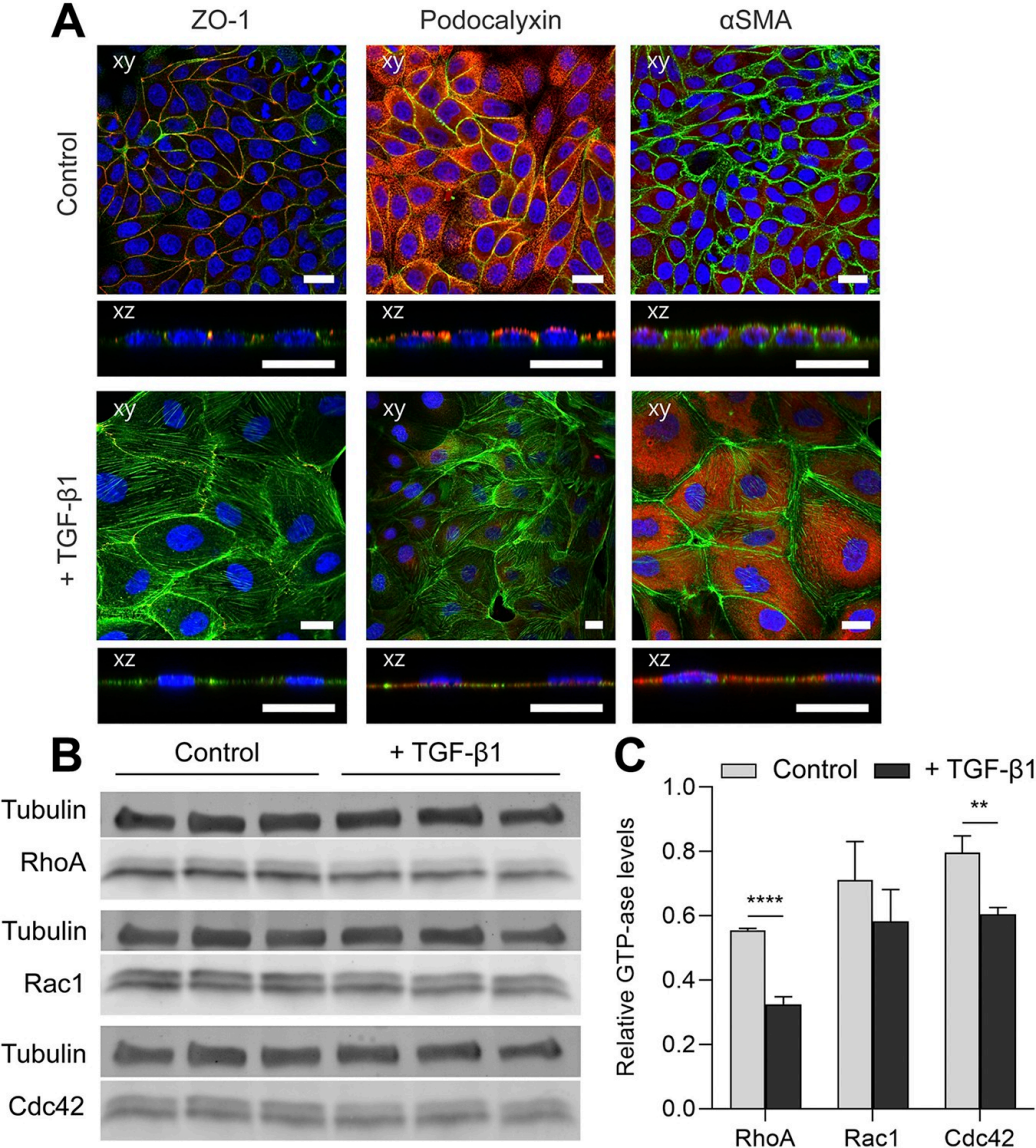
Expression of Rho-GTPases with and without the induction of EMT. (A) Samples were generally stained for F-actin (Phalloidin A488; green), nuclei (DAPI; blue), and for tight junctions (Zona Occludens 1 (ZO-1)), apical protein localization (Podocalyxin) or α-SMA expression (all in red). All scale bars are 20 μm. (B) Expression levels of RhoA, Rac1, Cdc42 and Tubulin as a loading control for three independent experiments were determined for MDCKS in the control condition as well as MDCKs exposed to TGF-β1 by western blotting using protein-specific antibodies. (C) Rho-GTPases bands were normalized to the loading control and the average relative densities for the control and for the cells exposed to TGF-β1 were calculated (error bars represent S.D., *n* = 3). (p-values were determined using a Student’s t-test, * p < 0.05, ** p < 0.01, *** p < 0.001, **** p < 0.0001).

*In vitro*, EMT was induced in Madin Darby Canine Kidney cells (MDCKs) via overnight serum starvation after 3 days of culture on transwell filters and the subsequent addition of TGF-β1 to the culture medium in a final concentration of 10 ng ml^-1^ for 1 day. MDCKs were also cultured for 4 days on transwell filters under normal conditions as a control. The visual hallmarks of polarization are the formation of tight junctions at the apical side of the lateral membrane, the localization of the apical proteins, the cortical organization of the actin cytoskeleton, the diminishment of mesenchymal markers and an increase in cell height. The MDCKs in the control condition displayed a clear polarized organization, with tight junction formation at the apical side of the membrane (Fig 11A: ZO-1) and the localization of the apical proteins at the apical membrane (Fig 11A: Podocalyxin). Control cells also showed a low expression of α-SMA (Fig 11A: α-SMA), a well-known mesenchymal marker [56]. In contrast, MDCKs exposed to TGF-β1 had no tight junctions, substantially higher levels of actin formation, no localization of the apical proteins, and they displayed an upregulation of αSMA expression: all indicators of a mesenchymal phenotype. Culture lysates were obtained after 4 days of culture to determine the expression of the Rho-GTPases after the induction of EMT. The expression levels of RhoA, Rac1 and Cdc42 together with Tubulin as a loading control, were obtained using immunoblotting (Fig 11B). Quantification of the relative band intensities shows a sharp decrease in total RhoA, a small decrease in total Cdc42, and no significant change in total Rac1 after the induction of EMT (Fig 11C). When comparing these decreases in the levels of the Rho-GTPases to our computational results, they were found to be most similar to the case of full Rho degradation. This similarity therefore suggests that degradation of Rho may be one of the key mechanisms in the TGF-β1-induced process of EMT in renal epithelial cells.

## 4. Discussion

In this study, we aimed to create a deeper understanding of the establishment of apical-basal polarization in renal epithelial cells; a key process in kidney morphogenesis and for the establishment of metabolic kidney function. To this end, we developed a computational framework to investigate how the interactions between integrins, Rho-GTPases (Rho, Rac, Cdc42), and polarity complexes (Par, Scribble) can enable the emergence of renal epithelial polarization.

We showed that the hypothesized protein interactions in our model can capture the establishment of polarized distributions of Par and Scribble, which is a key feature of apical-basal polarization in renal epithelial cells. Subsequently, an MPSA was used to identify critical factors in the establishment of polarization in our model. Since the parameter values for the rate constants and concentrations in our computational framework are based on multiple other cell types, these values could differ considerably between cell types and in different environments. Therefore, we chose to vary a large set of these parameters (21 parameters) simultaneously in a range of 20–500% of the original parameter value.

Through this global sensitivity analysis of the pathway, we found that integrins are crucial for the initiation of polarization, as has been determined by multiple experimental studies [21,47,57]. This initiation by integrins in our model was established through the assumption that active integrins were restricted to the basal side and thereby caused the subsequent signaling cascade. In case of a 1D model, this can be justified as active integrins are generally found at the membrane that is connected to the ECM. When this model would be extended to a 2D geometry, the active integrins would be able to bind at different locations on the cell membrane that is connected to the ECM and form clusters [58,59]. Integrins are also known to respond to different ligands [58,59], which could yield a competition for the activation of integrins [60]. A future extension of our model to 2D will allow for investigating if and how the activation of integrins by different ligands at different locations at the cell membrane can affect the signaling cascades that lead to polarization. The extension to 2D would also allow for the modeling cell-cell interactions. Cellular junctions have been shown to be highly involved in renal epithelial polarization [4,61–63]. Junctional proteins, such as claudins and ZO-1, have for example been shown to serve as a fence that prevents the mixing of apical and basolateral proteins [64–66]. More specifically, the loss of tight junctions has been shown to diminish epithelial polarity [67].

We identified Rho and Rac as critical components for the establishment of polarization in our model. This is consistent with the observation of Yu et al. [68] who observed that a decrease in Rac1 activity, caused by integrin inhibition, led to an upregulation of active RhoA. This hyperactivation in RhoA subsequently prevented the establishment of polarization [68]. It has been well documented that the interactions between Rho and Rac also trigger additional cell signaling cascades involved in cytoskeletal organization and cellular adhesion [69–72]. As these two processes are heavily affected by integrin-mediated signals from the environment, future studies should aim at elucidating whether Rho- and Rac-regulated cytoskeletal organization and adhesion also play a role in the process of polarization.

Other experimental studies have shown that epithelial cells display signs of polarization within 3 hours after cell seeding on glass coverslips [73]. In our model, the distributions of the polarity proteins already reached steady-state after approximately 80 seconds, which reveals much faster dynamics than those observed experimentally. Although the time that cells need to attach to a substrate prior to polarization (in the range of hours [74]) should be considered in this comparison, the time scales needed to establish polarization in our model and experiments certainly differ orders of magnitude. The parameter values used in this study are probably not responsible for this substantial difference in time scale, as variations of 21 parameters in a range of 20%-500% of the original values and thus by orders of magnitude did not lead to large differences in the time needed for polarization to develop. The most reasonable explanation is that the establishment of polarization *in vitro* is dependent on multiple mechanisms, such as cellular adhesion, cytoskeletal remodeling, the transportation of proteins via Rab-GTPase controlled traffickin cellular adhesion [75], cytoskeletal remodeling [76], the transportation of proteins via Rab-GTPase controlled trafficking [73] and cellular junction formation. In our model we only take into account the polarized sorting of proteins. While the timescales of protein sorting have not yet been investigated for the process of polarization, protein sorting and Rho-GTPase dynamics in general have been shown to be in the timescale of seconds or in case of lipid transport in mere subseconds [77–79], which agree well with our computational results. The other processes within the establishment of polarization have significantly larger timespans, especially cellular adhesion, cytoskeletal remodelling and junction formation. While actin remodelling has a relatively fast turnover (seconds) [80], apical-basal polarization also involves the remodelling of microtubular network, which has a slower polymerization rate (minutes to hours) [81]. The formation of cellular junctions, the last step in the development of apical-basal polarization, has also been shown to have a larger timespan [82]. Furthermore, apical-basal polarization has been shown to be a rather sequential process, instead of a parallel process [5,83]. The notion that one process sets off another also lengthens the time needed to achieve full apical-basal polarization.

We used our computational framework to investigate the role of integrins in the establishment of apical-basal polarization by varying the initial concentration of active integrins that acts as the input signal of the model. The model predicted that polarized distributions of the polarity complexes Par and Scribble only emerge when the initial concentration of active integrins passes a certain threshold (18 μM). The finding that obtaining a polarized state in the cell requires a stimulus corresponds to the characteristics of a wave-pinning model. Reaction-diffusion systems with bistable kinetics are known to support traveling waves with a front that propagates [84]. Mass conservation of each of the Rho-GTPases, which corresponds to the constant total concentration of the Rho-GTPase in the cell, then stalls that wave to produce a polar pattern. These wave pinning systems have the property that in our case there can be either an unpolarized state with homogeneous distributions for the different proteins or a polarized ‘patterned’ state. While our model shows this characteristic, achieving polarized distributions in the protein concentrations is dependent on the model setup with the chosen set of parameter values, as displayed by the results of the MPSA.

The found threshold agrees qualitatively with the experimental results from previous studies where a strongly diminished integrin concentration was not able to initiate apical-basal polarization in a 3D environment [46–48]. On the other hand, our recent experimental work on MDCK polarization in a 2D environment has shown that cells cultured on substrates with a supraphysiological stiffness, which is associated with elevated levels of activated integrins as well as integrin clustering [33], remain unpolarized [75]. In our computational model, however, an increase of the amount of active integrins to the total number of integrins available in the domain, did not disable the development of polarized profiles of the polarity complexes Par and Scribble. This can be explained by the fact that we exclusively modeled the signaling cascade via the integrin-receptors that is necessary for the initiation of apical-basal polarization, while the inhibitory effect of the mechanotransduction pathway on polarization, which likely increases with substrate stiffness, was ignored [75]. To confirm this hypothesis, a dual role of the integrins in both the mechanotransduction pathway and the polarization pathway should be included in the model.

Our results suggest that manipulation of integrin signaling via, for example, biomaterials may be an attractive method in controlling epithelial polarization. Control of polarization could aid in the regulation of subsequent lumen formation for the purpose of tubular tissue engineering or in the enhancement of the vectorial transport necessary for kidney function. We specifically found that a minimum concentration of active integrins was necessary to catalyze the signaling cascade that leads to polarization, but the strength of polarization was independent of the active integrin concentration beyond this threshold. Translating this finding to an *in vitro* or an *in vivo* situation suggests that a sufficient concentration of bioactive ligands should be incorporated in biomaterials to activate sufficient integrins to trigger polarization.

Additionally, we used our computational framework to investigate if and how degradation or upregulation of Rho-GTPases can affect the polarized steady-state situation and thereby induce EMT. We observed that only the full degradation of either Rho or Cdc42, and not Rac, can lead to the depolarization of the polarity complexes Par and Scribble. Within our computational framework, Rac regulates the initiation of polarization but when the polarized state has been reached, degradation of Rac is not able to affect the polarity complexes. This is in contrast with other experimental findings that show that Rac can play a role in EMT via other downstream effectors (e.g., Smad2 or p38 MAPK) [85,86]. The role of Rac in EMT can therefore not be explained via the direct interactions between the Rho-GTPases, as is the case in our model. Finally, our experimental results show that EMT in renal epithelial cells is associated with a large decrease in Rho concentration and a small decrease in Cdc42 concentration, which mostly corresponds with our simulations in which Rho was completely degraded. In previous experimental studies, Rho has also been identified as a key player in the process of EMT, especially since Rho and Cdc42 together are responsible for the stabilization of tight junctions, and disassembly of these junctions is the first step of EMT [11,87,88]. However, other studies demonstrated that activation of Rho can also cause the induction of EMT via downstream effectors (e.g., actin cytoskeleton, Smad2) [54,55]. One of these studies showed that TGF-β1 induced EMT is accompanied by a sharp 5-fold increase of active Rho [54,55], which suggests that EMT requires a large upregulation in Rho activation. Our simulations were not able to capture this phenomenon and even indicated that upregulation of Rho led to a slight enhancement of the polarized state of the cell. The ability of our model to capture TGF-β1 induced EMT via degradation but not via upregulation of Rho could be explained by the manner in which TGF-β1 affects Rho that leads to the induction of EMT. In case of degradation, it has been shown that TGF-β1 causes the ubiquitination and thus inactivation of Rho at the tight junctions [67]. The inactivation of Rho at the tight junctions then causes phosphorylation of Par6 at the apical side of the cell and thereby the dissociation of the tight junctions, which is one of the first steps of EMT [67]. The upregulation of Rho via TGF-β1 on the other hand is not a direct link but the result of signaling cascades, such as the mechanosensitive Rho pathway [89,90]. In this pathway, the resulting upregulation in Rho causes the activation of ROCK and the formation of stress fibers, which is one of the last steps in EMT [89,90]. This notion again suggests that activation of the mechanotransduction machinery (for example by using stiff substrates) can pose an inhibitory effect on polarization. Our model only investigates the direct coupling between the Rho-GTPases and the polarity complexes and does not involve other signaling cascades that could lead to a loss of polarity and thereby EMT. To capture and understand the entire process of EMT, future studies should include other signaling cascades that explore the downstream effects of TGF-β1.

The results of our predictive model and experiments together suggest that EMT can be induced by full degradation of the Rho-GTPase Rho. Although limitations exist in the targeting of the Rho-GTPases, since these proteins lie at the foundation of many other cellular processes, they could be involved in potential therapeutics for reversing EMT. A first step towards targeting Rho was given by Das et al. [91], who displayed that EMT could be reversed by a combination of multiple inhibitors of which the Rho kinase (ROCK) inhibitor was a critical factor.

Although we developed the current computational framework to investigate the establishment of apical-basal polarization in renal epithelial cells, the framework could also be used for applications in other species and/or organ specific cell types since polarization is known to be an evolutionary conserved mechanism. For every different application the interactions have to be investigated first and can be adjusted accordingly. For example, the polarization of endothelial cells, essential for/in vascular morphogenesis, is known to be initiated by integrin-based adhesion and signaling as well, and similarly requires the activation of Cdc42 and Rac1 [92,93]. In future studies, the signaling pathway involved in polarization could also be coupled to a mechanical model to investigate the interplay between cell polarity and the mechanical regulation of cell shapes that lead to tissue deformations. In addition to coupling our signaling model to a mechanical model, our signaling model could also be included in agent-based models that include cell-cell interactions. These agent-based models could then be utilized to examine polarization in the context of collective cell behavior, for example in the process of lumen formation [94].

In summary, in this study we developed a 1D computational framework to investigate the establishment of apical-basal polarization in renal epithelial cells that is based on the crosstalk between Rho-GTPases. The results of the MPSA that we performed to identify critical factors in the establishment of polarization suggest that the sideways inhibition of Rac and Rho has a dominant role in the polarization process. Furthermore, our computational simulations show that the concentration of active integrins needs to exceed a certain threshold (18 μM in our model) to initiate renal epithelial polarization via Rac activation. Finally, our model predicts that degradation of the small G-protein Rho may be a key player in the induction of EMT, where only complete degradation of Rho is sufficient to disrupt the stable polarized state. These findings improve our understanding of the signaling cascades involved in the establishment and disruption of apical-basal polarity and provides handles for controlling polarization for engineering purposes.

## 5. Experimental materials and methods

### 5.1. Cell culture

Madin Darby Canine Kidney II cells (MDCK-II, ECACC, The Netherlands) were cultured and expanded in a standard cell culture incubator (37°C, 5% CO_2_) in Eagle’s Minimum Essential Medium (EMEM; Merck, Darmstadt, Germany) supplemented with 5% fetal bovine serum (FBS; Greiner Bio-one, Alphen aan de Rijn, Netherlands), 1% penicillin/streptomycin (Invitrogen, Waltham, MA, USA), and 1% L-glutamine (Thermofisher Scientific, Waltham, MA, USA). Culture medium was refreshed every 3–4 days and the cells were passaged at 80% confluency. Cells were seeded onto transwell inserts (Nunc, Thermofisher Scientific, Waltham, MA, USA) at a density of 20,000 cells cm^-2^ for immunoprecipitation and immunofluorescence analysis. To induce EMT, after three days, cells were serum starved overnight and subsequently cultured in the presence of TGF-β1 (Peprotech EC Ltd., London, UK) in the medium in a final concentration of 10 ng mL^-1^ for the subsequent day. Culture of MDCKs for 4 days on transwell filters without EMT induction served as a control.

### 5.2. Western blot

MDCKs were seeded onto 3.1 cm transwell inserts (Thermofisher Scientific, Waltham, MA, USA) at a density of 20,000 cells cm^-2^. After four days, all samples were washed with ice-cold phosphate buffered saline (PBS) and incubated in lysis buffer (Cytoskeleton, Denver, CO, USA) for 10 minutes. After scraping, lysate was collected in Eppendorf tubes and centrifuged for 1 minute at 10,000xg at 4°C. Samples were sonicated and boiled for 5 min at 98°C for analysis with SDS-Page (4–15% gradient gel, Biorad, Lunteren, the Netherlands) and subsequent Western nitrocellulose blot (Amersham). In addition to loading 27 μg of the total protein samples, 106.5 pg of control protein RhoA and Cdc42 and 22.5 pg of control protein Rac1 were loaded. Membranes were blocked with 5% BSA/5% non-skim dry milk in PBS/0.05% Tween-20 and incubated (O/N, 4°C) with primary antibodies: mouse monoclonal anti-Cdc42 (1:500, Santa-Cruz Biotechnology, Dallas, TX, USA), mouse monoclonal anti-Rac1 (1:1000, Merck, Darmstadt, Germany), mouse monoclonal anti-RhoA (1:1000, Santa-Cruz Biotechnology, Dallas, TX, USA) and rat monoclonal anti-tubulin (1:5000, Novus Biologicals, Littleton, CO, USA). Secondary antibodies applied were Goat-anti-mouse-HRP (1:10,000, Invitrogen, Waltham, MA, USA) or Goat-anti-rat-Alexa633 (1:5000, Thermofisher, Waltham, MA, USA) for 2 hours at RT. Proteins were visualized using Supersignal West Atto ECL substrate and the iBright FL1500 Imaging system (Thermofisher, Waltham, MA, USA). Band intensities were analyzed using ImageJ software [95].

### 5.3. Immunofluorescence staining

MDCKs were seeded onto 4.1 cm transwell inserts. After four days, all samples were fixed with 3.7% formaldehyde solution (Merck, Darmstadt, Germany) in PBS for 15 minutes at room temperature (RT) four days after seeding and washed three times with PBS. The membrane was cut out of the chamber and cut in four. Samples were subsequently permeabilized with a 0.5% Triton-X-100 (Merck, Darmstadt, Germany) in PBS for 5 minutes at RT and blocked for 1 hour at RT in PBS with 10% horse serum to prevent nonspecific antibody binding. Samples were then stained with the primary antibodies overnight at 4°C in PBS with 1% horse serum. Primary antibodies used were mouse monoclonal anti-α-SMA (1:600; Dako, Glostrup, Denmark), mouse monoclonal anti-podocalyxin (1:200; Merck, Darmstadt, Germany) or the conjugated Zona Occludens 1 antibody (1:200; Thermofisher Scientific, Waltham, MA, USA). After washing three times with PBS, the cells were incubated with secondary antibodies in PBS for 1h at RT. During secondary antibody incubation, samples were also stained with Phalloidin Atto 488 (Merck, Darmstadt, Germany), which stains for F-actin. Samples were washed again three times with PBS and subsequently incubated with a 4’-6-diamidino-2-phenylindole solution (DAPI; Merck, Darmstadt, Germany) in PBS, staining for nuclei. Cells were finally washed three times with PBS and mounted with Mowiol (Merck, Darmstadt, Germany). Images were acquired using a Leica SP8 microscope with oil 63x magnification.

### 5.4. Statistical analysis

All experimental data were presented as the mean ± standard deviation. Data were tested for normality using the Shapiro Wilk test. Since data were normally distributed according to the Shapiro Wilk test, comparisons between two groups were analyzed using an independent two-tailed Student’s *t* test (GraphPad, La Jolla, CA, USA). Differences were considered as statistically significant when P<0.05.

## Supporting information

S1 Fig Integrins provide the directional cue for the establishment of polarization. Final steady-state distribution of the active forms of the Rho-GTPases (A/C) and of the formed polarity complexes Par and Scribble (B/D) in case of active integrin localization at *x* = 0 (A/B) and at *x* = 10 (C/D). The Par complex accumulates at the opposite membrane from the location of the active integrins and the Scribble complex accumulates at the same membrane as the active integrins.
(TIF)
