# Supplementary figures and images for: A computational analysis of the role of integrins and Rho-GTPases in the emergence and disruption of apical-basal polarization in renal epithelial cells
Source: PLoS Comput Biol. 2024 May 20;20(5):e1012140. doi: 10.1371/journal.pcbi.1012140 (PMC11142725; doi:10.1371/journal.pcbi.1012140)

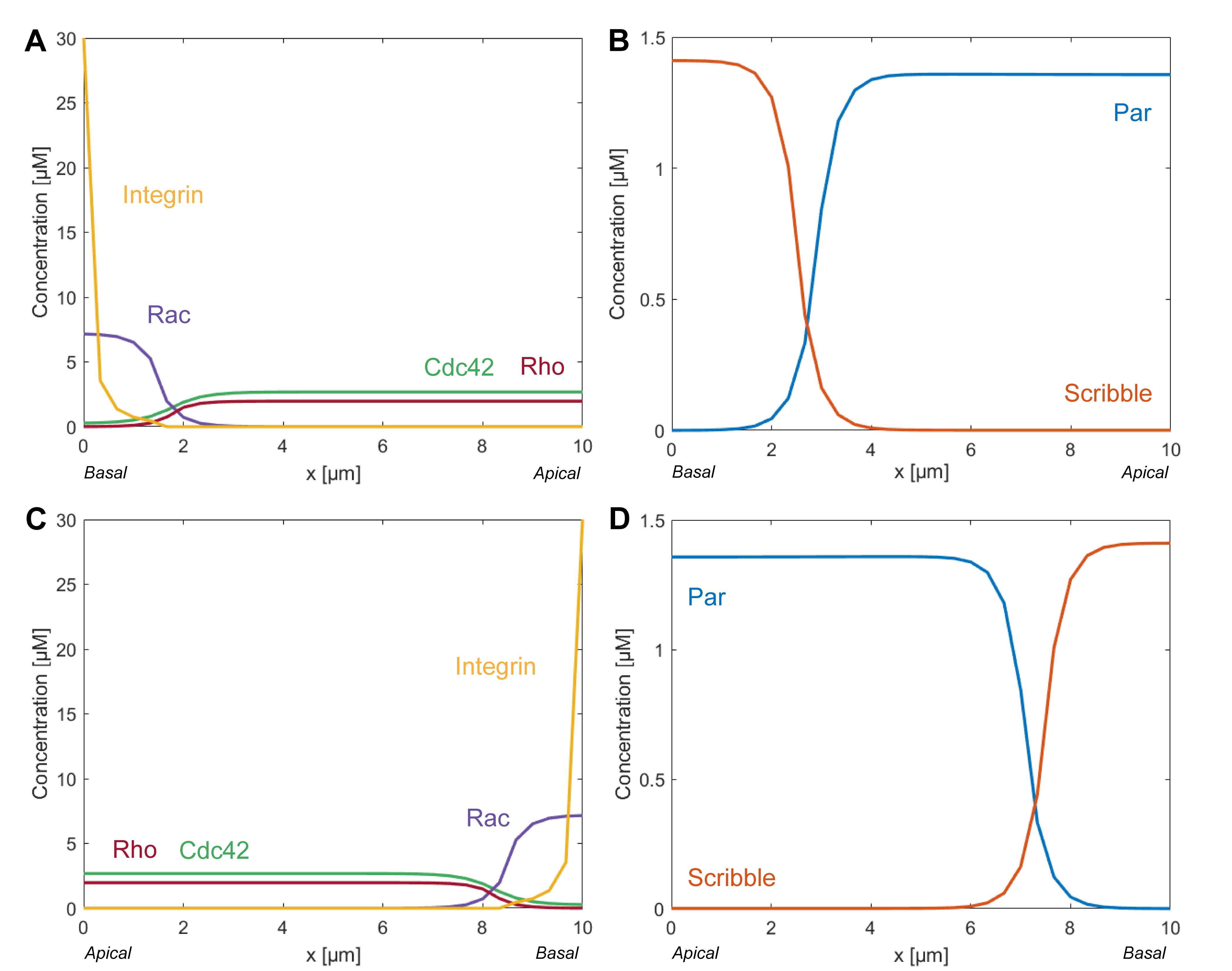

Supplement: S1 Fig — Final steady-state distribution of the active forms of the Rho-GTPases (A/C) and of the formed polarity complexes Par and Scribble (B/D) in case of active integrin localization at x = 0 (A/B) and at x = 10 (C/D). The Par complex accumulates at the opposite membrane from the location of the active integrins and the Scribble complex accumulates at the same membrane as the active integrins. (TIF) [file pcbi.1012140.s001.tif]
